# Gene expression analysis reveals early dysregulation of disease pathways and links *Chmp7* to pathogenesis of spinal and bulbar muscular atrophy

**DOI:** 10.1038/s41598-019-40118-3

**Published:** 2019-03-05

**Authors:** Bilal Malik, Helen Devine, Rickie Patani, Albert R. La Spada, Michael G. Hanna, Linda Greensmith

**Affiliations:** 1 Department of Neuromuscular Diseases, UCL Queen Square Institute of Neurology, Queen Square, London, WC1N 3BG UK; 2 MRC Centre for Neuromuscular Diseases, UCL Queen Square Institute of Neurology, Queen Square, London, WC1N 3BG UK; 3 The Francis Crick Institute, 1 Midland Road, London, NW1 1AT UK; 4 Department of Neurology, Duke University School of Medicine, Durham, USA; 5 Department of Neurobiology, Duke University School of Medicine, Durham, USA; 6 Department of Cell Biology, Duke University School of Medicine, Durham, USA; 7 Duke Centre for Neurodegeneration & Neurotherapeutics, Duke University School of Medicine, Durham, USA; 8 Department of Neuroscience, University of California, San Diego, La Jolla, USA; 9 Department of Cellular & Molecular Medicine, University of California, San Diego, La Jolla, USA; 10 Division of Biological Sciences, University of California, San Diego, La Jolla, USA

## Abstract

Spinal and bulbar muscular atrophy (SBMA) results from a CAG repeat expansion within the androgen receptor gene (*AR)*. It is unclear why motor neurons selectively degenerate and there are currently no treatments for this debilitating disease. To uncover the causative genes and pathways involved in motor neuron dysfunction, we undertook transcriptomic profiling of primary embryonic motor neurons from SBMA mice. We show that transcriptional dysregulation occurs early during development in SBMA motor neurons. One gene found to be dysregulated, *Chmp7*, was also altered *in vivo* in spinal cord before symptom onset in SBMA mice, and crucially in motor neuron precursor cells derived from SBMA patient stem cells, suggesting that *Chmp7* may play a causal role in disease pathogenesis by disrupting the endosome-lysosome system. Furthermore, genes were enriched in SBMA motor neurons in several key pathways including p53, DNA repair, WNT and mitochondrial function. SBMA embryonic motor neurons also displayed dysfunctional mitochondria along with DNA damage, possibly resulting from DNA repair gene dysregulation and/or mitochondrial dysfunction. This indicates that a coordinated dysregulation of multiple pathways leads to development of SBMA. Importantly, our findings suggest that the identified pathways and genes, in particular *Chmp7*, may serve as potential therapeutic targets in SBMA.

## Introduction

Spinal and bulbar muscular atrophy (SBMA), also known as Kennedy’s Disease, is a slowly progressive, adult onset neuromuscular disease, which primarily affects males. SBMA is characterised by degeneration of spinal and bulbar motor neurons, as well as fasciculation and atrophy of skeletal muscle, particularly in facial, bulbar and limb muscles^1^. In addition to the neuromuscular phenotype, patients may also present with mild sensory impairment and signs of androgen insensitivity, which manifests as gynaecomastia, testicular atrophy and reduced fertility. SBMA belongs to a group of inherited neurodegenerative diseases that result from an expansion of the polyglutamine (polyQ) encoding trinucleotide CAG repeat region in the coding region of causative genes^2^. Polyglutamine repeat expansion disorders also include Huntington’s disease, spinocerebellar ataxias (SCA1, 2, 3, 6, 7, and 17) and dentatorubralpallidoluysian atrophy. In SBMA, the expansion of the CAG repeat occurs in the first exon of the androgen receptor (*AR*) gene^3^. The polymorphic CAG repeat normally ranges from 9–36, while patients possess greater than 37 repeats.

Despite the ubiquitous expression of the causative *AR* gene and mutant protein, there is as yet no clear explanation for the selective loss of lower motor neurons in the anterior horn of the spinal cord and specific brainstem motor nuclei in SBMA, although high expression of *AR* within these cell types may be a possible contributory factor. Several pathomechanisims are thought to be involved in development of disease including ligand-dependent nuclear translocation^4–6^, protein misfolding and clearance^7,8^ as well as ER stress^9^. More recently, evidence has emerged that skeletal muscle may also play a critical role in disease pathogenesis^10,11^. However, there is a clear neurogenic contribution to the motor dysfunction observed in mice, suggesting the polyQ-expanded AR in motor neurons causes secondary pathology in muscle and is required for the development of the full range of symptoms^12^. Dysregulation of transcription plays a major role in the development of SBMA^7,13–17^. The steroid hormone AR is a ligand-inducible transcription factor which is activated following ligand (testosterone) binding. The expanded AR can also disrupt transcription by sequestering transcriptional factors and co-activators within inclusions formed by the pathogenic protein^18^. In order to identify candidate genes and molecular pathways involved in early motor neuron dysfunction, we undertook a global transcriptomic analysis of cultured embryonic motor neurons of the AR100 mouse model of SBMA. AR100 mice have a 100 CAG repeat expansion in the *AR* gene and develop a late-onset, gender (male) specific, progressive neuromuscular phenotype accompanied by motor neuron degeneration and muscle atrophy, which closely resembles patient symptoms^14,17,19^. Importantly, as no effective treatment or disease modifying therapies are available, the discovery of targets linked with early motor neuron dysfunction may provide promising therapeutic avenues in alleviating the development and course of the disease.

In this study, we found that the polyQ expansion in the AR results in transcriptional dysregulation which occurs very early in development and is present even in embryonic motor neurons from SBMA mice. *Chmp7*, which plays a role in autophagic flux and the endosome-lysosome system as part of the ESCRT-III complex, was altered in the analysis performed on embryonic motor neurons, as well as in adult mice *in vivo*, before symptom onset in the two primary sites of pathology, both in laser captured spinal cord motor neurons and hindlimb muscle, indicating the potential importance of this gene in disease pathogenesis. Significantly, we also found that *CHMP7* was downregulated in a SBMA human cell model derived from induced pluripotent stem cell (iPSCs) reprogrammed from patient fibroblasts to generate patterned ventral spinal cord motor neuron precursor cells (pMNs). *Chmp7* dysregulation was specific to SBMA, as the pathological change was absent in other forms of motor neuron disease (MND). In addition, we found that crucial biological pathways including p53, WNT, mitochondrial depolarisation and DNA repair, may be associated with the development of SBMA. In parallel, there was a decrease in mitochondrial, as well as antioxidant genes, resulting in abnormal mitochondrial membrane depolarisation, indicating mitochondrial dysfunction in embryonic motor neurons from AR100 mice. There were also signs of DNA damage in spinal cord motor neurons of AR100 mice, which may result from downregulation of DNA repair genes and/or mitochondrial dysfunction. The identified pathways and genes, particularly *Chmp7*, may therefore represent attractive molecular targets for development of a therapeutic approach for SBMA.

## Results

### Transcriptomic profiling of SBMA embryonic motor neurons

**Figure 1:**
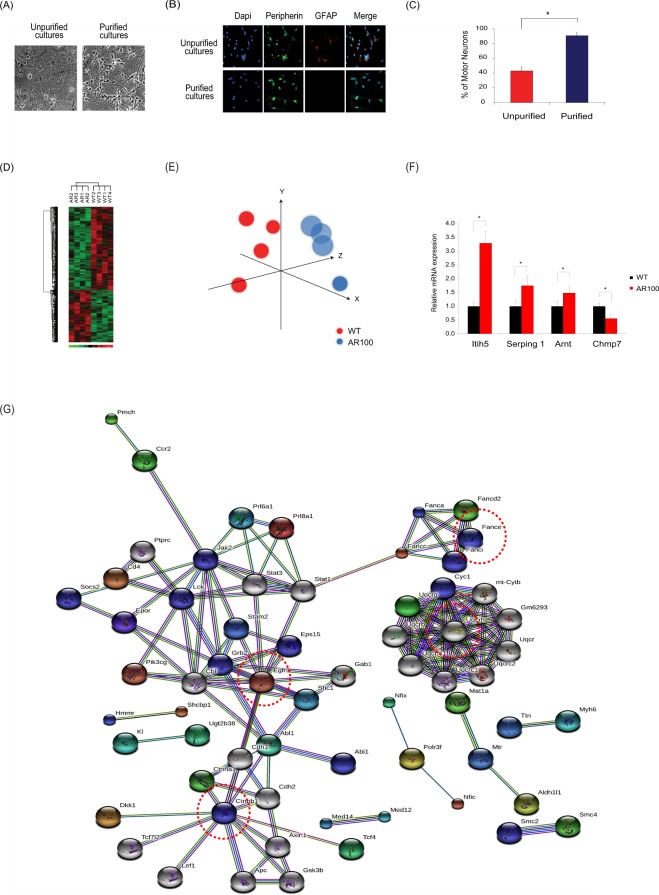
Transcriptomic profiling and gene expression analysis of primary motor neurons of SBMA mice. (**A**) Phase contrast pictures of purification of motor neuron cultures using Optiprep density centrifugation. (**B**) Immunostaining of cultures with peripherin (a marker of motor neurons in ventral horn cultures) and GFAP (astrocytes) was used to establish the number of motor neurons in culture. (**C**) Cell counts of cultures confirmed that 90% of cells are labelled as motor neurons after the purification protocol. (**D**) Cultured embryonic motor neurons treated with DHT from SBMA and wild-type (WT) mice have distinct transcriptional profiles. Principal component analysis (PCA) of gene expression data groups WT profiles together and distinct from AR100 data. (**E**) Similar findings were observed with hierarchical clustering of significant genes. (**F**) TaqMan qPCR was performed to determine expression of mRNA expression of indicated genes. *Itih5, Serping 1*, *Arnt* and *Chmp7* were dysregulated in AR100 DHT treated motor neurons relative to WT (n > 3, *P < 0.05). Data are displayed as mean ± SEM and are representative of independent experiments from three replicate cultures. Statistical analysis was performed using a two sample t-test (n ≥ 3, *P < 0.05). (**G**) String protein-protein network (PPI) was built using the significant gene list. The network was built around EGFR, UQCRC1, FANCE and CTNNB1 (circled by red dotted line). The generated networks represent associations between proteins. The colour of the lines represents different categories of evidence: neighbourhood in the genome (dark green line), gene fusion (red line), co-occurrence across genomes (dark blue line), co-expression (black line), experimental/biochemical data (purple line), association in curated databases (light blue line) and occurrence in text mining evidence (light green line).

To characterise early transcriptional dysregulation and establish disease mechanisms in SBMA, we first performed a global transcriptomic screen of purified cultured spinal cord motor neurons from embryonic AR100 and wild-type (WT) mice treated with dihydrotestosterone (DHT), to reflect the ligand dependency of the disease (Fig. 1A–C). We found that 178 genes were upregulated, whilst 287 genes were significantly downregulated in AR100 motor neurons compared with WT cultures (Supplementary Information, Fig. S1A–C and Tables S1, S2). Principal component analysis and hierarchical clustering discriminated between the WT and AR100 transcriptomes (Fig. 1D,E). The expression of several genes was verified using quantitative PCR (qPCR) (Fig. 1F and Supplementary Information, Fig. S1A). Upregulated genes included *Itih5* (inter-alpha-trypsin inhibitor heavy chain family member 5), which is involved in extracellular matrix stabilisation, and *Serping1* (serine protease inhibitor, C1-inhibitor of the complement system). The transcription factor *Arnt* (aryl hydrocarbon receptor nuclear translocator), otherwise known as *Hif1β*, a *Hif1α* co-factor, was also increased. *Chmp7* (Charged Multivesicular Body Protein7) was downregulated and is associated with ESCRT-III (the endosomal sorting complex required for transport) and generation of multivesicular bodies. Crucially, these genes were unaltered in control cultures prepared from the meninges or astrocytes of AR100 mice (Supplementary Information, Fig. S1D–F). Therefore, gene expression changes were specific to AR100 embryonic motor neurons. We also examined over-represented transcription factor binding sites in our differentially regulated genes using oPOSSUM^20^. Several sites were associated with FOXO signalling and regulation of oxidative stress (Supplementary Information, Table S3).

### Protein-protein interaction (PPI) network analysis

The differentially expressed genes were examined further using the STRING database. The resulting PPI network (Fig. 1G) centred on EGFR (epidermal growth factor receptor, a receptor tyrosine kinase of the ErbB family), UQCRC1 (ubiquinol-cytochrome c reductase core protein I, part of the mitochondrial respiratory chain), FANCE (Fanconi anemia complementation group E, functions in DNA repair) and CTNNB1 (catenin (cadherin-associated protein) beta 1), a downstream component of the canonical WNT signalling pathway. Furthermore, proteins/genes were enriched in several KEGG Pathways including: Huntington’s disease, Alzheimer’s disease, Parkinson’s disease, ErbB signalling pathway, adherens junction, WNT signalling pathway, focal adhesion, tight junction, neurotrophin signalling pathway, insulin signalling pathway and endocytosis (Table 1). Genes associated with Huntington’s, Parkinson’s and Alzheimer’s disease were all mitochondrial genes. Several of the protein/genes were enriched in the mitochondria in the GO cellular component analysis (Table 1), underlining the role mitochondria may play in the development of pathology in SBMA. Interestingly, many of the identified pathways were associated with axonal branching as well as neuromuscular junction formation and maintenance (focal adhesion, extracellular matrix, integrins, ErbB, endocytosis and WNT signalling). These findings suggest that the aberrant development and maintenance of neuromuscular junctions may be an early sign of pathology in the neuromuscular system of AR100 mice.

**Table 1:** Enrichment analysis of the STRING data.

ID	Term	Genes	p-value	Adjusted p-value
	**KEGG ID**			
mmu04260	Cardiac muscle contraction	13	7.48E-11	1.47E-08
mmu05213	Endometrial cancer	11	1.26E-10	1.47E-08
mmu05200	Pathways in cancer	19	2.31E-07	1.80E-05
mmu00190	Oxidative phosphorylation	12	4.89E-07	2.85E-05
mmu05210	Colorectal cancer	8	2.24E-06	1.04E-04
mmu05215	Prostate cancer	9	5.21E-06	2.02E-04
mmu04520	Adherens junction	8	8.30E-06	2.76E-04
mmu05412	Arrhythmogenic right ventricular cardiomyopathy (ARVC)	8	1.14E-05	3.21E-04
mmu05016	Huntington’s disease	12	1.24E-05	3.21E-04
mmu05217	Basal cell carcinoma	7	1.50E-05	3.49E-04
mmu05012	Parkinson’s disease	10	2.22E-05	4.71E-04
mmu04012	ErbB signaling pathway	8	2.46E-05	4.78E-04
mmu05010	Alzheimer’s disease	11	3.92E-05	7.03E-04
mmu04310	Wnt signaling pathway	10	6.01E-05	9.46E-04
mmu04510	Focal adhesion	10	6.04E-04	7.41E-03
mmu04910	Insulin signaling pathway	6	1.28E-02	8.37E-02
mmu04144	Endocytosis	8	1.28E-02	8.37E-02
mmu01100	Metabolic pathways	26	1.66E-02	9.94E-02
	**GO Cellular Component**			
GO:0005750	mitochondrial respiratory chain complex III	6	9.39E-12	6.75E-09
GO:0070069	cytochrome complex	6	8.06E-09	2.90E-06
GO:0070469	respiratory chain	10	4.03E-08	9.65E-06
GO:0016342	catenin complex	4	3.01E-06	6.18E-04
GO:0005912	adherens junction	12	8.23E-06	1.14E-03
GO:0030877	beta-catenin destruction complex	4	8.82E-06	1.14E-03
GO:0030054	cell junction	30	9.55E-06	1.14E-03
GO:0005743	mitochondrial inner membrane	16	3.47E-05	2.17E-03
GO:0005746	mitochondrial respiratory chain	6	9.71E-05	5.17E-03
GO:0031988	membrane-bounded vesicle	64	1.83E-04	8.23E-03
GO:0070369	beta-catenin-TCF7L2 complex	2	2.13E-04	9.30E-03
GO:0031966	mitochondrial membrane	17	2.99E-04	1.16E-02
GO:0065010	extracellular membrane-bounded organelle	54	3.14E-04	1.16E-02
GO:0005740	mitochondrial envelope	17	5.59E-04	1.75E-02
GO:0071664	catenin-TCF7L2 complex	2	6.34E-04	1.82E-02
GO:1990204	oxidoreductase complex	6	7.54E-04	2.05E-02
GO:0044455	mitochondrial membrane part	8	8.17E-04	2.14E-02
GO:0005768	endosome	15	3.15E-03	6.75E-02
GO:0030139	endocytic vesicle	6	4.09E-03	8.41E-02
GO:0005925	focal adhesion	6	4.29E-03	8.69E-02

### Gene ontology classification and Gene Set Enrichment Analysis

The differentially regulated genes were classified according to their biological process, molecular function and cellular component gene ontology (GO) terms (Supplementary Information, Fig. S2). Additional functional network and pathway annotation using ClueGO/CluePedia^21^ indicated that WNT, PI3K, insulin signalling pathways, mitochondrial depolarisation and intracellular trafficking may be disrupted in AR100 motor neurons (Table 2 and Supplementary Information, Fig. S3A) and furthermore, that several miRNA’s (mmu-miR-124-3p, mmu-miR-290-3p) may potentially interact with these pathways (Supplementary Information, Fig. S3B). Gene set enrichment analysis (GSEA) revealed that genes in the integrin pathway, extracellular matrix, p38MAP kinase and GSK3 signalling pathways (Table 3), as well as p53 signalling and lysosome function (Supplementary Information, Fig. S4A–C), were enriched in AR100 motor neurons compared with WT. Interestingly, the only gene set under-represented in AR100 motor neurons was the “neuroactive ligand receptor activation” KEGG pathway, in which the leading genes encoded glutamate receptors. This pathway is involved in synapse and neuromuscular junction development.

**Table 2:** Cluepedia enrichment of ClueGO protein-protein interaction network.

GO ID	Ontology Source	GO Term	% Associated Genes	Adjusted p-value
GO:0051901	GO Biological Process	positive regulation of mitochondrial depolarization	37.500	0.008
GO:0035358	GO Biological Process	regulation of peroxisome proliferator activated receptor signaling pathway	18.182	0.022
GO:2000505	GO Biological Process	regulation of energy homeostasis	15.385	0.026
GO:0002090	GO Biological Process	regulation of receptor internalization	11.111	0.018
GO:0035567	GO Biological Process	non-canonical Wnt signaling pathway	10.526	0.011
GO:0050848	GO Biological Process	regulation of calcium-mediated signaling	7.500	0.029
GO:0031397	GO Biological Process	negative regulation of protein ubiquitination	6.122	0.038
GO:0046824	GO Biological Process	positive regulation of nucleocytoplasmic transport	6.024	0.017
GO:0006282	GO Biological Process	regulation of DNA repair	5.556	0.045
GO:0007043	GO Biological Process	cell-cell junction assembly	5.556	0.045
GO:0031929	GO Biological Process	TOR signaling	5.556	0.045
GO:0051897	GO Biological Process	positive regulation of protein kinase B signaling	5.063	0.034
GO:0051896	GO Biological Process	regulation of protein kinase B signaling	5.042	0.017
GO:0030177	GO Biological Process	positive regulation of Wnt signaling pathway	4.494	0.041
GO:0032388	GO Biological Process	positive regulation of intracellular transport	4.420	0.014
GO:0051222	GO Biological Process	positive regulation of protein transport	4.348	0.009
GO:0005758	GO Cellular Component	mitochondrial intermembrane space	5.455	0.046
GO:0019199	GO Molecular Function	transmembrane receptor protein kinase activity	4.819	0.036
KEGG:04012	KEGG	ErbB signaling pathway	4.598	0.039
KEGG:04512	KEGG	ECM-receptor interaction	4.545	0.040
REACTOME:5892997	REACTOME	Methylation	20.000	0.020
REACTOME:5893278	REACTOME	mTORC1-mediated signalling	10.526	0.038
REACTOME:5892940	REACTOME	IGF1R signaling cascade	4.082	0.050

**Table 3:** GSEA analysis.

Name	Size	ES	NES	p-value	Adjusted p-value
**(A) Pathways enriched in AR 100 motor neuron**					
KEGG ECM receptor interaction	75	0.680	2.364	0	0
KEGG cell cycle	111	0.628	2.348	0	0
KEGG focal adhesion	180	0.570	2.260	0	0
KEGG DNA replication	32	0.682	2.066	0	0
KEGG small cell lung cancer	79	0.576	1.989	0	0.002
KEGG p53 signalling pathway	60	0.577	1.975	0	0.002
KEGG regulation of actin cytoskeleton	187	0.478	1.883	0	0.008
KEGG pathways in cancer	305	0.455	1.883	0	0.007
KEGG lysosome	109	0.490	1.817	0	0.015
KEGG lysine degradation	42	0.548	1.742	0.002	0.031
KEGG basal cell carcinoma	54	0.518	1.699	0.003	0.045
BioCarta GSK3 pathway	27	0.676	1.942	0.002	0.028
BioCarta cell cycle pathway	21	0.696	1.899	0	0.027
BioCarta G1 pathway	26	0.660	1.885	0	0.021
BioCarta integrin pathway	38	0.598	1.825	0	0.042
BioCarta ALK pathway	35	0.598	1.825	0.002	0.034
BioCarta p38 MAPK pathway	38	0.594	1.819	0	0.030
BioCarta HIV nef pathway	53	0.545	1.806	0	0.030
**(B) Pathways decreased in AR 100 motor neurons**					
KEGG neuroactive ligand receptor interaction	22	−0.427	−1.936	0	0.007
KEGG regulation of autophagy	24	−0.493	−1.504	0.038	0.302
KEGG autoimmune thyroid disease	23	−0.487	−1.447	0.045	0.305

### *Chmp7* is dysregulated in SBMA mice and human iPSC-derived pMNs

**Figure 2:**
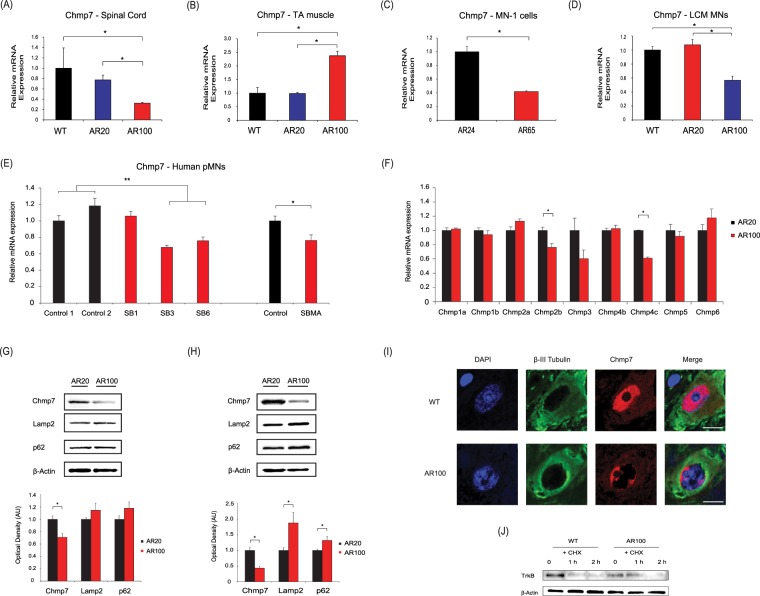
*Chmp7 is* differentially regulated in in presymptomatic SBMA mice and motor neuron precursor cells derived from SBMA patients. (**A**) *Chmp7* was downregulated in AR100 mice spinal cord compared to control WT and AR20 presymptomatic male mice at 3 months of age. (**B**) *Chmp7* expression was upregulated in tibialis anterior (TA) muscle of presymptomatic AR100. (**C**) C*hmp7* was also downregulated in the SBMA AR65Q neuronal stable cell line model and (**D**) in laser captured (LCM) ventral spinal cord motor neurons from presymptomatic AR100 mice. (**E**) *Chmp2b* was downregulated in AR100 mice, while *Chmp4c* was increased. qPCR data are displayed as mean ± SEM and are representative of at least three independent experiments. Statistical analysis was performed using a two sample t-test or one-way ANOVA followed by the Student–Newman–Keuls and Tukey’s Honestly Significantly Different *post hoc* tests (n ≥ 3, *P < 0.05). (**E**) *CHMP7* was downregulated in SBMA patient-derived motor neuron precursor cells (pMN). (*P < 0.05, **P < 0.01, two sample t-test or ANOVA with Tukey’s Honestly Significantly Different *post hoc* test). (**G**) The protein levels of Chmp7, Lamp2 and p62 were analysed by Western blot of spinal cord of AR100 and control AR20 male mice. Chmp7 was decreased in presymptomatic (3 month) AR100 mice. (**H**) Chmp7 was further reduced in symptomatic (12 month) AR100 mice, while Lamp2 levels were increased. Densitometric analysis of bands was performed using values normalized to actin. Data are displayed as mean ± SEM and are representative of three independent experiments. Statistical analysis was performed using a two sample t-test (n ≥ 3, *P < 0.05). AU = arbitrary units. (**I**) CHMP7 staining in motor neurons of 3 month old presymptomatic WT and AR100 mice. β-III tubulin was used to stain motor neurons. Scale bars represent 20 μm. (**J**) TrkB degradation was performed using primary motor neuron cultures starved for 1 h in serum-free medium without BDNF. Cells were incubated with BDNF and cycloheximide (CHX). In AR100 cultures there was a delay in the degradation of TrkB compared to WT.

We next analysed the expression of *Chmp7* in the spinal cord as well as the tibialis anterior muscle of male mice at different stages of disease progression, as they represent the primary sites of pathology in SBMA (Fig. 2). We found the following striking results: in presymptomatic AR100 mice (3 month spinal cord), there was a 2.74 fold decrease in *Chmp7* (AR100 vs AR20, n = 3, *P < 0.01) (Fig. 2A). AR20 mice carry 20 polyQ repeats and therefore lack any phenotype and are indistinguishable from WT mice^14,19^. In contrast, there was a 2.5 fold increase in *Chmp7* in tibialis anterior of AR100 mice (AR100 vs AR20, n = 3, *P < 0.01) (Fig. 2B). There was no difference in *Chmp7* expression between WT and AR20 mice. *Chmp7* expression was also found to be decreased in MN-1 AR65Q cells (Fig. 2C), and crucially, in laser capture microdissection (LCM) derived motor neurons isolated from spinal cord of presymptomatic AR100 mice (Fig. 2D), emphasising a key role of this gene in the early dysfunction of motor neurons. We next examined the expression of other members of the CHMP family in the spinal cord of presymptomatic AR100 mice (Fig. 2E). There was downregulation of *Chmp2b* (1.34 fold decrease, n = 3, *P < 0.05) in AR100 mice relative to AR20 mice, as well as a decrease in *Chmp4c* (1.64 fold decrease, n = 3, *P < 0.05). We also analysed *Chmp7* expression in the cortex of presymptomatic AR100 mice and found mRNA levels were unchanged (Supplementary Information, Fig. S5). Interestingly, when *Chmp7* expression was examined in the spinal cord of presymptomatic mice from two separate mouse models of MND, the SOD1-G93A mouse model of Amyotrophic Lateral Sclerosis (ALS) and the SETX-R2136H model of ALS4, an adolescent-onset form of ALS, we found no significant differences between WT and mutant mice (Supplementary Information, Fig. S5). Taken together, these findings suggest that *Chmp7* dysregulation is specific to SBMA and is not present in (at least two) other forms of MND, and is predominantly confined to the two primary sites of pathology in SBMA, i.e. spinal cord motor neurons and hindlimb muscle. In addition, when we examined the expression of *CHMP7* in a human SBMA cell model generated from iPSC derived pMNs generated from SBMA patients, we found a significant decrease in two of the three SBMA lines studied (SB3, 1.47 fold decrease, **P < 0.01; SB5, 1.32 fold decrease, **P < 0.01), compared to the two unaffected control lines (Fig. 2F).

As part of the ESCRT-III complex, CHMP7 sorts ubiquitinated proteins from endosomes to the lysosome through formation of multivesicular bodies and delivers autophagosomes to lysosomes (autophagic flux). We therefore examined the expression of autophagy-related genes in AR100 mice and found that although *Chmp7* was decreased in the spinal cord, there was no significant change in autophagy associated genes (*p62*, *Lc3b, Lamp1, Lamp2, Atg5, Atg7, Atg12*) in presymptomatic AR100 mice (Supplementary Information, Fig. S6A). However, in the spinal cord of symptomatic AR100 mice there was an increase in *Lc3b* mRNA expression (Supplementary Information, Fig. S6B), which may be indicative of a disruption in autophagic flux.

In the spinal cord of 3 month old AR100 presymptomatic mice, CHMP7 protein was decreased (1.4 fold decrease, n > 3, *P < 0.05) (Fig. 2G and Supplementary Information, Fig. S7), but levels of Lamp2 and p62 (linked with autophagic protein degradation in the lysosome) were unchanged compared to control AR20 mice. After the onset of symptoms in AR100 mice, CHMP7 was markedly decreased (2.3 fold decrease, n > 3, *P < 0.05), but the levels of LAMP2 (1.9 fold increase, n > 3, *P < 0.05) and p62 protein were elevated (1.3 fold increase, n > 3, *P < 0.05) (Fig. 2H and Fig. S7). Unfortunately, immunostaining for LAMP2 in the spinal cord proved inconclusive (Supplementary Information, Fig. S6C). These changes therefore suggest there is a possible disruption of autophagic flux in the spinal cord of AR100 mice, due to ESCRT-III complex related *Chmp7* dysregulation, leading to increased LAMP2 and p62.

Furthermore, immunostaining revealed that CHMP7 displayed a diffuse cytoplasmic and nuclear localisation in WT spinal cord motor neurons (Fig. 2I and Supplementary Information, Fig. S8). However, in presymptomatic AR100 motor neurons, CHMP7 expression was localised to the perinuclear region with a possible change in the nucleus/nucleolus ratio. When we examined the degradation of TrkB in cultured motor neurons, we found that in AR100 cultures the rate of TrkB degradation was slower compared to WT, suggesting that CHMP7 dysregulation may be associated with disruption of the endosomal-lysosomal degradation pathway and receptor recycling in SBMA AR100 mice (Fig. 2J and Supplementary Information, Fig. S7).

### Mitochondrial dysfunction in embryonic SBMA motor neurons

**Figure 3:**
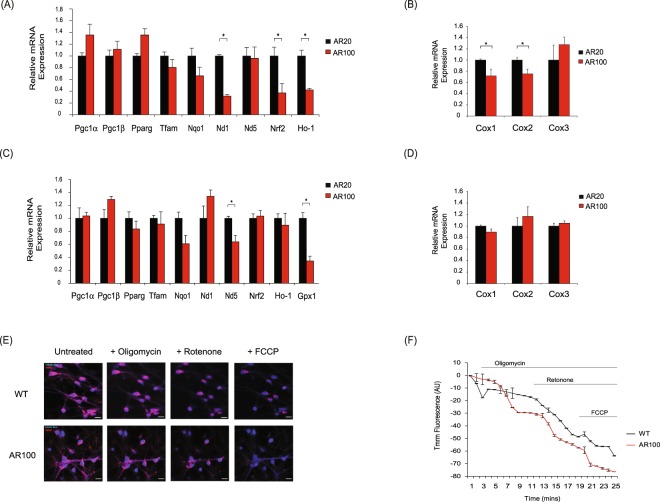
Dysregulation of mitochondrial genes and disruption of mitochondrial membrane potential in SBMA mice. (**A**) The expression of mitochondrial associated genes was established by qPCR in E13 AR100 and control AR20 motor neurons cultures. (**B**) The expression of cytochrome c oxidase subunit genes, *Cox1* and *Cox2* was reduced in AR100 E13 motor neurons, with no significant change in *Cox3*. (**C**) Mitochondrial genes were also examined in spinal cord of 3 month old presymptomatic AR100 and control AR20 mice. (**D**) *Cox1*, *Cox2* and *Cox3* mRNA expression was not altered in AR100 presymptomatic mice. Data are displayed as mean ± SEM and are representative of three independent experiments. Statistical analysis was performed using a two sample t-test (n ≥ 3, *P < 0.05). (**E**) Mitochondrial membrane potential (ΔΨm) of embryonic motor neurons treated with DHT was determined by live cell imaging of AR100 and AR20 motor neurons using TMRM. Live cell imaging was performed by recording images continuously from a single focal plane. Mitochondria were loaded with TMRM and motor neurons were labelled with 1 nM calcein-blue AM. (**F**) The effect of mitochondrial toxins (oligomycin, rotenone and FCCP) was measured in a time series experiment. AR100 motor neurons became strongly depolarised after addition of the toxins. Scale bars represent 10 µM. Data are displayed as mean ± SEM and are representative of at least three independent experiments. Statistical analysis was performed using a repeated measures ANOVA followed by the Bonferroni *post hoc* test (n ≥ 4, *P < 0.05). AU = arbitrary units.

Our bioinformatic pathway and network analysis predicted that mitochondrial genes may be dysregulated in motor neurons of AR100 mice. We therefore investigated the expression of mitochondrial associated genes using qPCR. *ND1* (NADH dehydrogenase), as well as the cytochrome c oxidase subunit genes, *Cox1* and *Cox2* were all downregulated (Fig. 3A,B). Additionally, the antioxidant gene *Ho-1* (heme oxygenase-1) was decreased, as was the *Nrf2* factor (nuclear factor erythroid 2 transcription), which regulates the expression of antioxidant genes. In the spinal cord of AR100 presymptomatic mice, *ND5* (NADH dehydrogenase 5) was reduced compared to AR20 mice, along with the antioxidant gene *Gpx1* (glutathione peroxidase 1) (Fig. 3C,D). *ND1* and *ND5* transcribe proteins which are part of oxidative phosphorylation active mitochondrial complex I. In all cases the data was similar between AR20 and WT cultures and mice (data not shown). Given the abnormal expression of mitochondrial genes detected in cultured AR100 motor neurons, we examined the mitochondrial membrane potential (ΔΨM), an indicator of mitochondrial health, using live cell imaging and TMRM fluorescence intensity. Cultures exposed to DHT were treated over a time course with the mitochondrial toxins oligomycin (binds ATP synthase to inhibit oxidative phosphorylation), rotenone (inhibits the electron transport of complex I) and FCCP, a protonophore uncoupling agent (Fig. 3E,F). Following oligomycin treatment, which causes hyperpolarisation of the ΔΨM (control cultures), there was no increase in TMRM fluorescence in AR100 motor neurons, but a gradual decrease in fluorescence intensity. Exposure to rotenone accelerated the decline in TMRM fluorescence in AR100 motor neurons compared to control, indicative of an abnormally enhanced depolarisation of ΔΨM. Treatment with FCCP at the end of the experiment resulted in lower levels of TMRM fluorescence in AR100 motor neurons relative to control cells, reflecting an increased depolarisation of ΔΨM. These findings suggest that embryonic motor neurons of AR100 mice are more susceptible to ΔΨM defects following exposure to mitochondrial toxins than control cells.

### Dysregulation of p53 associated DNA repair and WNT pathway genes

**Figure 4:**
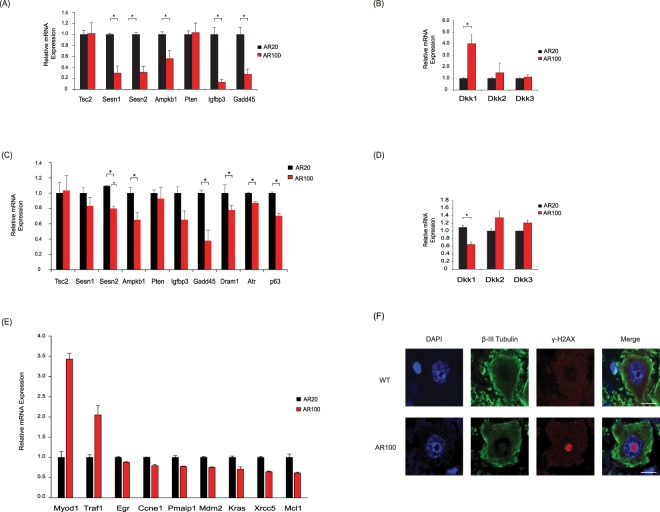
Dysregulation of *p53* associated DNA repair and WNT genes and DNA damage in SBMA mice. (**A**) The mRNA expression of *p53* target genes tuberous sclerosis complex 2 (*Tsc2*), sestrin 1 (*Sesn1)*, sestrin 2 (*Sesn2*), AMP-activated protein kinase (*Ampkb*), phosphatase and tensin homolog (*Pten*), IGF-binding protein 3 (*Igfbp3*) and growth arrest and DNA damage-inducible 45 (*Gadd45*) was examined in E13 AR100 and control AR20 motor neurons cultures. *Sesn1, Sesn2, Ampkb, Igfbp3, Gadd45 and Ho-1* were downregulated. (**B**) The WNT inhibitory gene *Dkk1* was increased, with no change in *Dkk2* and *Dkk3* expression. (**C**) The expression of *p53* target genes *Sesn2, Ampkb, Igfbp1, Gadd45, Dram1* and *p53* associated genes *Tp63* (p63) and *Atr* was downregulated in spinal cord of AR100 relative to control AR20 presymptomatic mice, at 3 months of age. (**D**) *Dkk1* was downregulated in presymptomatic AR100 mice. qPCR data are displayed as mean ± SEM and are representative of three independent experiments. Statistical analysis was performed using a two sample t-test (n ≥ 3, *P < 0.05). (**E**) A p53 PCR array was used to determine the expression of genes associated with the p53 pathway from spinal cord of AR20 and AR100 presymptomatic mice of 3 months of age. *Myod1* and *Traf1* were increased, while *Egfr*, *Ccne1*, *Pmaip1*, *Mdm2*, *Kras*, *Xrcc5* and *Mcl1* were significantly downregulated. qPCR data are displayed as mean ± SEM. Statistical analysis was performed using a two sample t-test (*P < 0.05). (**F**) Ventral horn spinal cord was immunostained with γ-H2AX (a marker of DNA damage) and β-III tubulin . There was evidence of γ-H2AX foci in motor neurons of 12 month old AR100 mice. Scale bars represent 10 μm.

GSEA and PPI network analysis also suggested that the p53 and WNT pathways may be dysregulated. In cultured embryonic motor neurons from AR100 mice, the p53 target genes sestrin 1 (*Sesn1)*, sestrin 2 (*Sesn2*), AMP-activated protein kinase (*Ampkb*), IGF-binding protein 3 (*Igfbp3*) and growth arrest and DNA damage-inducible 45 (*Gadd45*) were all decreased (Fig. 4A). These genes function in different facets of the p53 signalling pathway; DNA repair and damage (*Sesn1, Sesn2, Gadd45, Igfbp3, Ampkb)*, suppression of reactive oxygen species and protection from oxidative stress (*Sesn1, Sesn2)*, IGF (*Igfbp3)*, mTOR (mammalian target of rapamycin) and autophagy pathway (*Sesn1, Sesn2, Ampkb)*. Dickkopf-1 (*Dkk1)*, a p53 target gene and an important inhibitor of the canonical WNT signalling pathway was increased (Fig. 4B). WNT signalling is vital in synapse formation and maintenance of synapse integrity^22,23^. Therefore, it is possible that changes in Dkk1 expression observed in AR100 motor neurons may mediate downstream deleterious effects on the neuromuscular junction. There was no difference in the expression of the examined genes between AR20 and WT cultures and mice (data not shown). In spinal cord of presymptomatic AR100 mice, *Sesn2*, *Ampkb, Igfbp3* and *Gadd45* were downregulated, and additionally *Dram1* was decreased (Fig. 4C). *Tp63* (p63*)*, which belongs to the *p53* family of gene, and the *p53* activator *Atr* (ataxia-telangiectasia mutated and Rad3-related) were also decreased and are associated with the DNA damage and repair response. For all genes, results were similar between AR20 and WT mice (data not shown).

*Dkk1* expression decreased, in contrast to the finding observed in embryonic motor neurons (Fig. 4D). Furthermore, *p53* targets, *Gpx1* (spinal cord) and *Ho-1* (embryonic motor neurons) were downregulated (Fig. 3A,C). We next used a PCR array plate with p53 target genes and found that in the spinal cord of presymptomatic AR100 mice, *Myod1* (Myogenic Differentiation 1) and *Traf1* (TNF receptor-associated factor 1) were increased, while *Egfr* (epidermal growth factor receptor), *Ccne1* (cyclin E1), *Pmaip1* (phorbol-12-myristate-13-acetate-induced protein 1), *Mdm2* (transformed mouse 3T3 cell double minute 2), *Kras* (Kirsten rat sarcoma viral oncogene homolog), *Xrcc5* (X-ray repair complementing defective repair in Chinese hamster cells 5) and *Mcl1* (myeloid cell leukemia sequence 1) were downregulated (Fig. 4E). Several of the *p53* associated and *p53* target genes impact on DNA damage response and repair (*Sesn1, Sesn2*, *Gadd45, Egfr*, *Kras*, *Xrcc5, Tp63* and *Atr*). Importantly, following the downregulation of DNA damage/repair genes we found signs of DNA damage in AR100 mouse spinal cord, implying the motor neurons are vulnerable to this type of damage as a result of compromised repair processes. Histone H2AX is a marker of double stranded DNA breaks and is phosphorylated at S139 (γ-H2AX) in the presence of DNA damage^24^. γ-H2AX was found in the nuclei of motor neurons of symptomatic AR100 mice (Fig. 4F), suggesting that DNA damage may have a role in SBMA.

**Figure 5:**
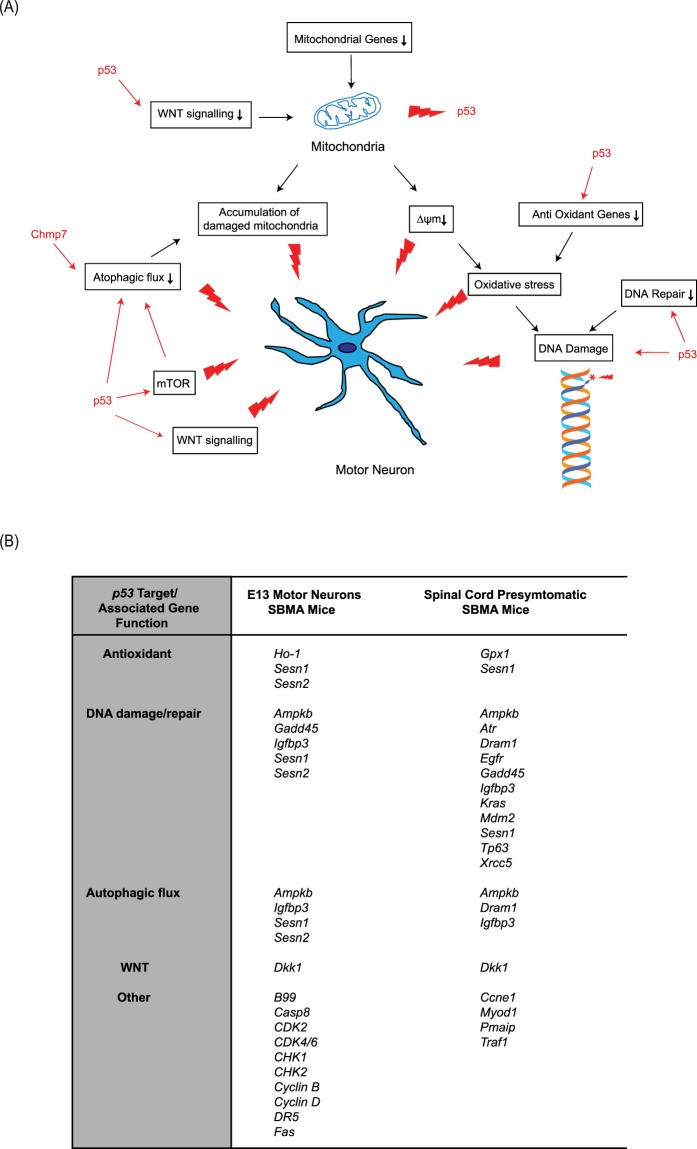
The role of p53 and mitochondria in pathogenesis of SBMA. (**A**) The role and interaction of *p53* and mitochondria in pathways to degeneration in SBMA motor neurons. (**B**) *p53* targets in E13 embryonic motor neurons and spinal cord from presymptomatic SBMA AR100 mice.

In summary, as shown in Fig. 5, our results predict that along with mitochondrial dysfunction, alterations in the p53 pathway, may be important early components of disease in SBMA.

## Discussion

To uncover targets directly responsible for motor neuron pathology in SBMA which are altered prior to the onset of symptoms, rather than dysregulated as a result of the disease, we undertook a transcriptomic analysis of cultured embryonic motor neurons of AR100 SBMA mice. We found that transcriptional dysregulation occurs very early in development. One of the dysregulated genes, *Chmp7*, was also altered in adult AR100 mice in the key tissues affected by disease, and these changes occurred before symptom onset, indicating the potential importance of this gene in the development of disease. *Chmp7* dysregulation displayed disease specificity as it was altered in SBMA but the pathological change was absent in other forms of MND. Crucially, we also found that *CHMP7* was downregulated in human SBMA patient iPSC-derived pMNs. The identified genes were enriched in multiple pathways (p53, DNA repair, WNT, mitochondrial depolarisation), and in particular mitochondrial and p53 dysfunction may be important drivers of disease. Our results, summarised in Fig. 5, highlight the importance of the interplay of multiple pathways in the pathogenesis of SBMA and the likelihood that this early coordinated dysregulation, in particular *Chmp7* dysfunction plays a critical role in the development of disease.

*Chmp7* may play a key role in the development of SBMA, with evidence of dysregulation at the two primary sites of pathology, spinal cord and muscle, observed in AR100 mice both *in vitro* and *in vivo*. Importantly, we also found a significant downregulation in *CHMP7* in a human stem cell-derived model of SBMA. As the changes in *Chmp7* expression were detected before the onset of symptoms, it is likely that this dysregulation in *Chmp7* expression plays a role in the initiation of pathology, rather than being a consequence of disease. Several *Chmp* genes were downregulated in the spinal cord (*Chmp7, Chmp2b, Chmp4c*), suggesting a coordinated malfunction in this family of genes which may serve as primary drivers of disease. Interestingly, downregulation of *Chmp7* expression was not present in other models of MND, the SOD1-G93A ALS mouse model and the SETX-R2136H model of ALS4. It suggests that the identified pathological dysregulation of *Chmp7* is perhaps specific to SBMA. CHMP7 is a CHMP4-associated ESCRT-III related protein^25^ and functions in the endosomal sorting pathway by shuttling ubiquitinated proteins from endosomes to the lysosome through formation of multivesicular bodies^26^. Accumulation of ubiquitinated proteins in the perinuclear area was reported in HeLa cells expressing CHMP7, as well as inhibition in levels of endocytosed EGF^25^. The CHMP family of proteins therefore influence receptor recycling and control of signalling pathways such as EGF and neurotrophins^27^. Dysregulation of CHMP/ESCRT complex may also affect mitochondria clearance^28^. Additionally, the *Chmp7* related ESCRT complex is linked with synapse maintenance^29^ and synaptic transmission^30^. Mutations in *CHMP2B* are known to be a cause of MND, indicating that any Chmp7 related dysfunction may be critical in the development of SBMA^31^. *Chmp7* dysregulation in SBMA AR100 mice may not only disrupt the endosomal-lysosomal degradation pathway and receptor recycling, but along with other members of the *Chmp* family of genes, may also be mechanistically responsible for abnormalities in autophagic flux which have previously been detected in AR100 mice^7^. Our results show CHMP7 protein levels were decreased in the spinal cord of AR100 mice prior to symptom onset and decreased further following symptom onset. LAMP2 and p62 were elevated, as was *Lc3b* expression, but only in symptomatic mice, which may signify an increase in lysosomes and autophagosomes, as a consequence of CHMP7/ESCRT-III complex related disruption of the lysosomal degradation of autophagosomes. *Chmp7* was also altered in hindlimb muscles of presymptomatic AR100 mice, although in contrast to spinal cord, expression in muscle was increased. Preliminary findings suggest that at this early stage of disease, there may be an initiation of pathways controlling muscle atrophy (data not shown) and as a consequence, muscle attempts to compensate and regenerate, resulting in an increase in endocytosis, membrane trafficking and autophagic flux (downstream of *Chmp7* increase), as a response to the initiation of atrophy and degeneration programmes.

Although our results suggest that several pathways may be associated with SBMA pathogenesis, it is possible that mitochondrial and p53 dysfunction are the key, early drivers of disease (Fig. 5A,B). Mitochondrial deficits and p53 dysfunction may act independently and in parallel, but also synergistically to initiate the early features of disease. However, whether one pathway is upstream of the other remains unclear. It is possible that expanded AR-associated transcriptional dysregulation disrupts mitochondrial gene expression, resulting in abnormal ΔΨM, leading to the development of oxidative stress and eventual DNA damage. Mitochondrial dysfunction may be an early driver of disease as it may affect *p53*-mediated transcription^32^, which can simultaneously downregulate antioxidant and DNA repair genes, further escalating oxidative stress and DNA damage. A reciprocal effect of *p53* on mitochondria may also occur, as DKK1 may disrupt mitochondrial biogenesis and mitochondrial DNA content in primary fibroblasts and muscle cells^33^. Therefore, it is also conceivable that *p53* dysfunction is the early instigator of disease. However, the possibility remains that *p53* may independently and directly target antioxidant genes and DNA repair genes, not as a consequence of mitochondrial dysfunction.

Several *p53-*associated DNA damage response/repair genes were dysregulated in the spinal cord of AR100 mice (Fig. 5B): *Sesn1* and *Sesn2*, which induce antioxidant genes, but also mediate the DNA damage response^34^; *Gadd45*, which functions in DNA nucleotide excision repair^35^; EGFR which regulates DNA repair^36^; KRAS, which is involved in the DNA damage response as a downstream component of the EGFR pathway^37^; *Xrcc5*, which encodes Ku80, and together with Ku70 forms the Ku heterodimer, which binds to DNA double-strand breaks and has a role in the non-homologous end joining pathway of DNA repair^38^; IGFBP3, which inhibits IGF-1/AKT signalling, and which also functions in DNA repair, as it forms a complex with the EGFR, translocating to the nucleus to interact with DNA-dependent protein kinase^39^; *Tp63* (p63) and *Atr* are associated with DNA damage repair^40,41^. These findings suggest that early insults to embryonic motor neurons, such as mitochondrial dysfunction and repression of antioxidant genes, can lead to oxidative DNA damage occurring in conjunction with impairments in the protective response of DNA repair, triggering further pathological injury. Interestingly, the antioxidant gene regulator *Nrf2*, which was downregulated in the spinal cord of AR100 mice, can also interact with the p53 pathway and facilitate the DNA damage response^42^; therefore, its downregulation may have a detrimental impact on DNA damage repair. *Nrf2* has previously been shown to be associated with SBMA^43^. The loss of DNA repair function can render motor neurons vulnerable to further insult, such as oxidative stress, resulting in DNA damage. There is evidence of mitochondrial abnormalities in SBMA; Ranganathan *et al*. reported deficits in MN-1 cells and SBMA knock-in AR-113Q mice^44^, whilst mitochondrial irregularities have also been shown to be present in muscle of SBMA patients^45^. However, the results of this study predicts the interaction with other pathways such as *p53* in the development of pathology in SBMA. Crucially, we show that there may be two concurrent insults: mitochondrial gene dysregulation coupled with ΔΨM disruption and *p53*-mediated dysregulation of antioxidant and DNA damage/repair genes, which may diminish protection against mitochondrial dysfunction and DNA damage. These abnormalities occur early in development and may be key instigators of pathology in SBMA motor neurons.

*p53* dysfunction may be damaging to motor neurons through other mechanisms. *p53* leads to the inhibitory actions of *Sesn1*, *Sesn2*, *Ampkb1* on the mTOR pathway^46^. The mTOR pathway coordinates energy metabolism, particularly when oxidative phosphorylation is impaired^47^ inhibiting autophagy, leading to accumulation of damaged mitochondria. Therefore, downregulation of *Sesn1*, *Sesn2*, *Ampkb1* in AR100 motor neurons may result in a loss of *p53*-dependent inhibition of mTOR, mediating increased energy usage which, in turn, may not only intensify the mitochondrial deficits but may also reduce clearance of damaged mitochondria, causing increased cell stress. *p53* can also activate several genes such as DRAM1 and CHMP4C^48^, which mediate autophagosome clearance. *Dram1* plays an important role in autophagic flux, and ΔΨM dysfunction can act as a signal to limit further damage via autophagy^49^. Downregulation of this gene in AR100 mice would therefore potentially disrupt the clearance of damaged mitochondria.

Using an integrated systems biology approach, combining differential gene expression with pathway and network analysis, we sought to understand in this study the causative changes that result in motor neuron dysfunction in SBMA. Our findings suggest a complex interplay and crosstalk between several different pathogenic signalling pathways following polyQ-expanded AR-mediated transcriptional dysregulation in SBMA mice. Firstly and most important of all, the mechanistic failure of autophagic flux may be dependent on the dysregulation of the *Chmp* family of genes, including *Chmp7*, which may disrupt the endosomal-lysosomal degradation pathway as well as receptor recycling in SBMA. Secondly, several dysregulated genes converge on the p53 pathway and are associated with autophagic flux, the WNT pathway, antioxidant function and DNA damage response/repair. Finally, mitochondrial and p53 deficits may be early instigators of dysfunction possibly acting independently or also synergistically to initiate the early features of disease in motor neurons. Taken together, these findings indicate that an interplay of multiple pathways contribute to the disease pathogenesis of SBMA. Significantly, the dysregulated genes and pathways, and in particular *Chmp7/CHMP7* identified by our transcriptomic profiling may serve as candidate druggable molecular targets for therapy development in SBMA.

## Materials and Methods

### Animals

All experimental procedures were carried out under licence from the UK Home Office (Scientific Procedures Act 1986) and following approval by the UCL Institute of Neurology Animal Welfare Ethical Review Panel. Male mice carrying the AR with 100 (pathogenic AR100 mice) or 20 polyQ repeats (non-pathogenic AR20 mice) were mated with WT C57BL/6 J females and maintained at UCL Institute of Neurology Biological Services. SBMA AR100 mice carry 100 CAG repeats in the AR gene as determined by Sanger sequencing. Genotyping by PCR was performed as described^50^. In accordance with the gender specificity of the human disease, only male mice were used in this study. These experiments were designed to use the minimum number of animals required for reliable statistical analysis and the 3 R principles were considered at each stage of the experimental planning.

### Tissue collection

Muscle and spinal cord tissue was collected from WT, AR100 and AR20 control male mice at different stages of disease progression: presymptomatic mice (3 months old), symptomatic (12 months). For western blotting and quantitative PCR (qPCR), fresh spinal cord and muscles were removed and snap frozen. For cryosectioning of spinal cords, the mice were terminally anaesthetized with pentobarbitone and transcardially perfused with 4% paraformaldehyde. The spinal cords were removed, post-fixed with 4% paraformaldehyde and cryoprotected overnight with 30% sucrose. Spinal cord cross-sections were subsequently cut on a cryostat at 10 µm.

### Primary embryonic motor neuron cultures

Purified primary motor neuron cultures were prepared from the spinal cords of embryonic day 13 (E13) mice using Optiprep density gradient centrifugation as previously described^19,51,52^. Motor neurons were re-suspended in neurobasal medium containing 50 U/mL penicillin, 50 μg/mL streptomycin, 2% B27 supplement, 25 µM 2-mercaptoethanol, 2% horse serum and 0.5 mM L-glutamine (all Invitrogen), 0.1 ng/ml glial-derived neurotrophic factor, 0.1 ng/ml brain-derived neurotrophic factor and 0.5 ng/ml ciliary neurotrophic factor (all Peprotech). After 4 days the media was changed to neurobasal without horse serum. Cultures were treated with 50 nM DH for 3 days before use. Cultures were grown on poly-ornithine and laminin coated plates for 7 days in total and were maintained at 37 °C in 5% CO2 and 95% air. TrkB receptor assay is described in Supplementary Information.

### iPSC-derived patterned motor neuron precursor cells

iPSCs derived from SBMA patients were kindly provided by Dr Kurt Fischbeck’s lab (National Institute for Neurological Disorders and Stroke, USA). The generation and characterisation of iPSC-derived patterned ventral spinal cord motor neuron precursor cells (pMNs) used in this study have been described previously^53^. Two control and three SBMA (SB1, SB3, SB6) lines were used. pMNs were generated as described^54,55^. Additional details are described in Supplementary Information.

### MN-1 cells

MN-1 cells (mouse spinal cord motor neuron-derived hybrid) with either the full-length pathogenic AR with 65 glutamines (AR65Q) or the non-pathogenic AR with 24 glutamines (AR24Q) were grown as described^56^ and in Supplementary Information.

### Immunofluorescence

Motor neuron cultures were fixed with 4% (w/v) paraformaldehyde for 20 min at room temperature. Cells were permeabilised and blocked by addition of PBS containing 0.1% (w/v) Triton-X100 and 3% (v/v) milk and 5% serum (v/v) for 1 h. The following primary antibodies were used: Peripherin (1:500; Encor, USA), α-GFAP-Cy3 conjugated antibody (1:500; Sigma-Aldrich). AlexaFluor488 was used as the secondary antibody (Thermo Fisher Scientific). Nuclei were counterstained with DAPI and coverslips were mounted using fluorescent mounting medium (Dako). Images were acquired using a Leica DMR fluorescence microscope.

Serial 10 µm spinal cord transverse sections were blocked and permeabilised at room temperature for 1 h in PBS containing 0.1% (w/v) saponin and 5% serum (v/v) and incubated overnight at 4 °C with the following primary antibodies: β-III tubulin (1:500, Biolegend), CHMP7 (1:250, Sigma-Aldrich) Primary antibodies were detected using Alexa Fluor 488 or Alexa Fluor 568 secondary antibodies (Thermo Fisher Scientific) and nuclei were stained with DAPI (Sigma-Aldrich). Images were acquired using a Zeiss LSM 510 confocal microscope.

### Laser capture microdissection

Laser capture microdissection (LCM) was performed on cryostat cut spinal cord sections (15 µm), attached to RNase-free polyethylene naphthalate (PEN) membrane covered glass slides to facilitate laser cutting as described^52^. LCM was carried out with a PALM Zeiss Microbeam microscope using PALM LCM protocols to capture at least 1000 motor neurons per spinal cord onto CapSure Macro LCM caps (Arcturus Bioscience). RNA was extracted using the RNesy micro kit (Qiagen) according to the manufacturer’s instructions.

### Western blot

Western blot using mouse spinal cords was performed as described^57,58^. Proteins were separated by SDS-PAGE, transferred onto nitrocellulose membrane, incubated with indicated primary antibodies: CHMP7 (Santa Cruz), Lamp2 (Novus Biologicals), p62 and β-actin (Abcam); followed by horseradish peroxidase conjugated secondary antibodies (Dako). The blots were imaged using the ChemiDoc Touch Imaging System (BioRad) and densitometry was performed using the Image Lab (BioRad) and ImageJ software (NIH, Bethesda, USA).

### Measurement of mitochondrial membrane potential (∆ψm)

The mitochondrial membrane potential (∆ψm) of cultured primary motor neuron mitochondria treated with DHT was measured using 20 nM tetramethylrhodamine methyl ester (TMRM) (Thermo Fisher Scientific) in recording media (156 mM NaCl, 10 mM HEPES, 10 mM D-glucose, 3 mM KCl, 2 mM MgSO_4_, 2 mM CaCl_2_, 1.25 mM KH_2_PO_4_, pH 7.35) as described^59^. Calcein-blue AM (1 nM, Thermo Fisher Scientific) was used to label motor neurons. Images were taken on Zeiss Laser Scanning 780 confocal microscope (Carl Zeiss) using the ZEN LE Digital imaging software. TMRM intensity was measured using ZEN 2 lite (Carl Zeiss). Blinded time series experiments were performed using images recorded continuously from a single focal plane. To monitor mitochondrial toxin effect, 2.5 μg/ml of oligomycin was added, followed by 5 µM rotenone and finally 1 µM of FCCP (carbonyl cyanide p-[trifluoromethoxy]-phenyl-hydrazone). Data analysis was performed as described^60^. TMRM intensity was normalised to the baseline fluorescence (ΔF = F - Fo/Fo × 100, where F = fluorescence intensity at any time point, Fo = baseline fluorescence).

### RNA extraction and qPCR

Total RNA was extracted from motor neuron cultures and laser captured spinal cord motor neurons using the RNesy micro kit (Qiagen). Total RNA was extracted from spinal cord and muscle using TRIzol® (Invitrogen). cDNA was prepared using superscript III reverse transcriptase, according to manufacturer instructions (Invitrogen). Real-time qPCR was performed as described^50^, using the Applied Biosystems 7500 Real-Time PCR System and TaqMan or Power SYBR Green PCR mastermix (Applied Biosystems) with specific primers (Supplementary Information, Tables S4 and S5). Reactions were performed in triplicate and values normalised using the geometric mean of the housekeeping genes, β-actin, hypoxanthine guanine phosphoribosyl transferase-1 (*Hprt1*) or phosphoglycerate kinase 1 (*pgk1*)^61^. Housekeeping genes were chosen by using NormFinder^62^. Relative quantification of gene expression was calculated via the comparative threshold cycle (ddCt) method^63^.

### Gene expression analysis

Four biological replicate samples from purified primary motor neuron cultures treated with DHT were used for genome wide analysis using Affymetrix 430 v2.0 mouse arrays, according to manufacturer’s instructions (Affymetrix). Experiments were performed by UCL Genomics. Data was analysed using R and BioConductor. Data has been deposited at ArrayExpress (ArrayExpress accession E-MTAB-6986). The robust multichip average (RMA) algorithm was used to normalise the microarray data sets and the Simpleaffy package^64^ was run to assess the quality of the arrays. The empirical Bayes moderated t-statistics test from the Limma package^65^ was used to identify the differentially expressed genes. Hierarchically clustering of genes using the Spearman rank correlation and principal component analysis was performed using dChip and BrB-Array tools software.

### Gene ontology (GO) analysis and GO term enrichment

Functional enrichment analysis was performed using Cytoscape plug-ins ClueGO and Cluepedia^21,66^. ClueGO analyses functional interaction of gene using GO information and databases including, KEGG (Kyoto Encyclopedia of Genes and Genomes), Reactome and BioCarta. Two-sided hypergeometric statistic was performed with kappa score threshold setting ≥0.3. All other default parameters were used. Enrichment/depletion was calculated based on p-value corrected using the false discovery rate (FDR).

### Gene set enrichment analysis (GSEA)

Further investigation of gene functional pathways was performed using Gene Set Enrichment Analysis (http://www.broad.mit.edu/gsea/) as described^67^. The statistical significance of the ES scores was empirically estimated by performing 1,000 random permutations of the ranked gene list. The differentially expressed genes were assigned to the functional gene sets of GSEA, and enrichment of pathways was established using a significance of *P* < 0.05 and FDR < 0.05.

### STRING protein-protein interaction network

The differentially expressed genes were used as an input to build and attain direct and indirect protein-protein interactions using STRING 9.0 database (Search Tool for the Retrieval of Interacting Genes)^68^. This database provides information on both experimental and predicted interactions from varied sources based on their neighbourhood, gene fusions, co-occurrence, co-expression, experiments and literature mining. We constructed an extended network based on a high confidence score of 0.9, which implies that only interactions with highest level of confidence were extracted from the database and considered as valid links for the PPI network.

### Statistical analysis

Statistical analysis was performed using SPSS v20 (SPSS Inc., USA), with the unpaired Student’s t-test (two-tailed) or ANOVA with corresponding post hoc tests performed to determine significance of data (*P* < 0.05).

## Supplementary information

Supplementary Information

## Data Availability

Gene expression data has been deposited at ArrayExpress (E-MTAB-6986).
