# Supplementary material for: Gene expression analysis reveals early dysregulation of disease pathways and links Chmp7 to pathogenesis of spinal and bulbar muscular atrophy
Source: Sci Rep. 2019 Mar 5;9:3539. doi: 10.1038/s41598-019-40118-3 (PMC6401132; doi:10.1038/s41598-019-40118-3)

Supplementary Information

Gene expression analysis reveals early dysregulation of disease pathways and links *Chmp7* to pathogenesis of spinal and bulbar muscular atrophy

Bilal Malik[1, 2*], Helen Devine[1, 2], Albert R. La Spada[3, 4, 5, 6],

Michael G. Hanna[2, 7] and Linda Greensmith[1, 2*]

Supplementary Methods

Primary culture

Primary spinal cord astrocyte cultures were established as previously described [1]. Spinal cords were isolated from postnatal day zero (P0) to P2 transgenic mouse pups. Cells were plated on poly-D-lysine coated plates in DMEM media supplemented with 10% foetal bovine serum and 1% penicillin and streptomycin (all Invitrogen). Cells were maintained for up to 2 weeks to allow cells to become confluent at which point the O2A progenitor cells and microglia were dislodged and removed. Cultures were immunoreactive for the astrocytic marker glial fibrillary acidic protein (GFAP) and plates were maintained at 37°C in 5% CO2 and 95% air.

In order to ensure differentially expressed genes were specific to motor neurons, we established cells cultured from meninges (predominantly fibroblasts removed from spinal cords dissected from embryos used for primary motor neuron cultures). These cultures were grown on poly-ornithine and laminin coated plates for 7 days *in vitro* in motor neuron culture media: neurobasal medium with 50U/mL penicillin, 50 µg/mL streptomycin, 2% B27 supplement, 25 µM 2-mercaptoethanol, 2% horse serum and 0.5 mM L-glutamine (all

Invitrogen), 0.1 ng/ml glial-derived neurotrophic factor, 0.1 ng/ml brain-derived neurotrophic factor and 0.5 ng/ml ciliary neurotrophic factor (all Peprotech). Cultures were treated with 50 nM dihydrotestosterone for 3 days before use. Cultures were maintained 7 days *in vitro* (DIV) in total at 37°C in 5% CO2 and 95% air.

MN-1 cells

MN-1 cells were maintained in Dulbecco's Modified Eagle's Medium with 10% foetal bovine serum (Thermo Fisher Scientific), 2 mM-glutamine and 100 units/ml penicillin/streptomycin. All the experiments were carried out in complete medium containing 10% charcoal dextran stripped-foetal bovine serum (Sigma-Aldrich), medium supplemented with 350 mg/ml G418 (Thermo Fisher Scientific). Cells were treated with 10 nM DHT for 48h before use.

iPSC-derived patterned motor neuron precursor cells

Induced pluripotent stem cells (iPSCs) (SB1, SB3 and SB6) from SBMA patients were kindly provided by Dr Kurt Fischbeck's lab at the National Institute for Neurological Disorders and Stroke (USA) through a material transfer agreement (MTA). Control 1 was available from Dr Rickie Patani's lab, UCL, UK and was reprogrammed using transfection of episomal plasmids as previously reported. [2]. Control 2 is commercially available from Coriell (cat number ND41866*C). iPSCs were maintained on Matrigel (Corning) with Essential 8 Medium media (Life Technologies) for Ctrl1 and Ctrl2 and mTESR-1 (Stem Cell Technologies) for SB1, SB3 and SB6. They were passaged using EDTA (Life Technologies, 0.5mM). iPSCs were plated to 100% confluency and then differentiated into neuroepithelium in chemically defined medium consisting of DMEM/F12 Glutamax, Neurobasal, L-Glutamine, N2 supplement, non-essential amino acids, B27 supplement, β-mercaptoethanol (all from Life Technologies) and insulin (Sigma). Between day 0-7 cells were treated with

small molecules as follows: 1μM Dorsomorphin (Millipore), 2μM SB431542 (Tocris Bioscience) and 3.3 μM CHIR99021 (Miltenyi Biotec). At day 4 and day 12, the neuroepithelial layer was dissociated using dispase (GIBCO, 1 mg/ml) and re-plated on matrigel coated plates. From day 8 cells were patterned for 7 days with 0.5 μM retinoic acid and 1 μM purmorphomine. At day 14 spinal cord patterned motor neuron precursors (pMNs) were treated with 0.1 μM purmorphamine for a further 4 days. The precursors were harvested at day 18 for the experiments described in this paper. All cell cultures were maintained at 5% carbon dioxide and 37 C.

Total RNA was extracted from neural precursor cells cultures using the RNeasy mini kit (Qiagen). cDNA was prepared using superscript IV reverse transcriptase, according to manufacturer instructions (Thermo Fisher Scientific). qPCR was performed using TaqMan® Fast Advanced Master Mix (Thermo Fisher Scientific) with specific primers (Supplementary Information, Tables S5). Reactions were performed in triplicate and values normalised using β-actin (*Actb*). Relative quantification of gene expression was calculated via the comparative threshold cycle (ddCt) method [3].

TrkB receptor assay

For the TrkB degradation assay, primary motor neuron cultures were grown as described for 7 DIV and were starved for 1 h in serum-free neurobasal medium without the TrkB ligand, BDNF. Cells were then incubated for the indicated time points with BDNF (10 ng/ml) and with cycloheximide (1 μg/ml), which inhibits synthesis of new protein. Cells were harvested in RIPA buffer (20 mM Tris pH 7.5, 150 mM NaCl, 1% NP-40, 0.5% deoxycholate, 0.1% SDS, 1 mM EDTA, 1mM EGTA, Halt Protease and Phosphatase Inhibitor Cocktail from Thermofischer) and analysed using Western Blot with antibodies against TrkB and β-actin.

Animals

The SOD1-G93A model of Amyotrophic lateral sclerosis (ALS) harbouring a mutation in the *SOD1* gene is a widely used mouse model to recapitulate many of the features seen in ALS, the commonest form of motor neuron disease (MND) [4,5]. The generation and characterisation of the ALS4 SETX-R2136H mice carrying the R2136H mutation in the human SETX gene has been described previously [6]. Spinal cord from presymptomatic mice was used for qPCR analysis.

Gene Ontology (GO) analysis and GO Term Enrichment

Differentially regulated genes were annotated using Gene Ontology (GO) classification, using PANTHER (http://www.pantherdb.org/). The Gene Ontology (GO) project describes gene products in terms of their associated biological processes, cellular components and molecular functions [7]. We examined higher level GO terms using GO slim terms, which are cut-down versions of the GO ontologies containing a subset of the terms in the whole GO giving a broad overview of the ontology content without the detail of the specific fine grained terms. To further understand the biological relevant significantly differentially regulated genes, we performed functional enrichment analysis using Cytoscape plug-ins ClueGO and CluePedia [8,9]. ClueGO analyses functional interaction of gene using GO information and database including, KEGG (Kyoto Encyclopedia of Genes and Genomes) and Reactome gene functional databases (http://www.reactome.org/). KEGG is a database of biological systems that integrates genomic, chemical and systemic functional information [10-12]. BioCarta (http://www.biocarta.com/) provides useful pathway information. ClueGO creates the first binary gene-term matrix with the selected terms and their associated genes and term-term similarity matrix is calculated using chance corrected kappa statistics. The kappa score measures the association strength between overlapping GO terms. A threshold of 0.3 (i.e., GO term pairs with kappa score $\geq$ 0.3 are connected in the ClueGO network).

Enrichment/depletion was calculated based on p-value corrected using hypothesis testing correction using the false discovery rate (FDR) method of Benjamin and Hochberg [13] and Storey [14]. CluePedia provides further insights into pathways by integrating experimental and in silico information and extends ClueGO functionality down to genes and miRNAs [8]. CluePedia calculates statistical correlation between differentially expressed genes and integrated variables using Pearson correlation, Spearman's rank, Distance correlation and Minimal Information Criteria (MIC). This allows investigation of a pathway by combining known and new, experimental derived information, about genes/proteins involved such as miRNAs influencing the expression of the genes.

Transcription factor binding sites

oPOSSUM software (http://opossum.cisreg.ca/oPOSSUM3/) was used to identify transcription factor binding sites in the differentially regulated genes [15]. This allows identification of enriched potential transcription factor binding sites in promoter regions of genes. The mouse single site analysis (SSA) was used, with a 0.4 conservation cut-off and a matrix score threshold of 80% and by using by using 2000/0 on the upstream/downstream score.

Immunofluorescence

Serial 10 µm transverse spinal cord sections were used for immunofluorescence staining. Sections were blocked and permeabilised at room temperature for 1 h in PBS containing 0.1% (w/v) Triton-X100 (v/v) and 5% serum (v/v) and incubated overnight at 4ºC with the following primary antibodies: β-III tubulin (1:500, Biolegend), Chmp7 (1:250, Sigma UK), γ-H2AX (1:500, Millipore) and LAMP2 (1:250, Novus Biologicals) . Primary antibodies were detected with Alexa Fluor 488

References

1 Bilsland, L. G., Nirmalananthan, N., Yip, J., Greensmith, L. & Duchen, M. R. Expression of mutant SOD1 in astrocytes induces functional deficits in motoneuron mitochondria. *J. Neurochem* **107**, 1271-1283 (2008).

2 Hall, C. E. *et al.* Progressive Motor Neuron Pathology and the Role of Astrocytes in a Human Stem Cell Model of VCP-Related ALS. *Cell Rep* **19**, 1739-1749 (2017).

3 Livak, K. J. & Schmittgen, T. D. Analysis of relative gene expression data using real-time quantitative PCR and the 2(-Delta Delta C(T)) Method. *Methods* **25**, 402-408 (2001).

4 Kieran, D. *et al.* Treatment with arimoclomol, a coinducer of heat shock proteins, delays disease progression in ALS mice. *Nat. Med* **10**, 402-405 (2004).

5 Rosen, D. R. *et al.* Mutations in Cu/Zn superoxide dismutase gene are associated with familial amyotrophic lateral sclerosis. *Nature* **362**, 59-62 (1993).

6 Bennett, C. L. *et al.* Senataxin mutations elicit motor neuron degeneration phenotypes and yield TDP-43 mislocalization in ALS4 mice and human patients. *Acta Neuropathol* **136**, 425-443 (2018).

7 Ashburner, M. *et al.* Gene ontology: tool for the unification of biology. The Gene Ontology Consortium. *Nat. Genet* **25**, 25-29 (2000).

8 Bindea, G., Galon, J. & Mlecnik, B. CluePedia Cytoscape plugin: pathway insights using integrated experimental and in silico data. *Bioinformatics* **29**, 661-663 (2013).

9 Bindea, G. *et al.* ClueGO: a Cytoscape plug-in to decipher functionally grouped gene ontology and pathway annotation networks. *Bioinformatics* **25**, 1091-1093 (2009).

10 Kanehisa, M. & Goto, S. KEGG: kyoto encyclopedia of genes and genomes. *Nucleic Acids Res* **28**, 27-30 (2000).

11 Kanehisa, M., Furumichi, M., Tanabe, M., Sato, Y. & Morishima, K. KEGG: new perspectives on genomes, pathways, diseases and drugs. *Nucleic Acids Res* **45**, D353-D361 (2017).

12 Kanehisa, M., Sato, Y., Furumichi, M., Morishima, K. & Tanabe, M. New approach for understanding genome variations in KEGG. *Nucleic Acids Res* **47**, D590-D595 (2019).

13 Benjamini, Y. & Hochberg, Y. Controlling the False Discovery Rate: A Practical and Powerful Approach to Multiple Testing. *Journal of the Royal Statistical Society* **57**, 289-300 (1995).

14 Storey, J. D. & Tibshirani, R. Statistical significance for genomewide studies. *Proc. Natl. Acad. Sci. U. S. A* **100**, 9440-9445 (2003).

15 Ho Sui, S. J. *et al.* oPOSSUM: identification of over-represented transcription factor binding sites in co-expressed genes. *Nucleic Acids Res* **33**, 3154-3164 (2005).

Table S1. Transcripts upregulated in AR100 primary motor neurons

Probe Set ID	Gene Symbol	Gene Title	p-value	FC	GO biological process term
**1429159_at**	Itih5	inter-alpha (globulin) inhibitor H5	0.009	1.86	negative regulation of peptidase activity
**1428909_at**	A130040M12Rik	RIKEN cDNA A130040M12 gene	0.035	1.81	---
**1436755_at**	Itih5	inter-alpha (globulin) inhibitor H5	0.004	1.75	negative regulation of peptidase activity
**1448550_at**	Lbp	lipopolysaccharide binding protein	0.002	1.67	leukocyte chemotaxis involved in inflammatory response
**1441946_at**	Itih5	inter-alpha (globulin) inhibitor H5	0.026	1.55	negative regulation of peptidase activity
**1416625_at**	Serping1	serine (or cysteine) peptidase inhibitor, clade G, member 1	0.014	1.40	negative regulation of complement activation, lectin pathway
**1437760_at**	Galnt12	UDP-N-acetyl-alpha-D-galactosamine:polypeptide N-acetylgalactosaminyltransferase 12	0.044	1.37	carbohydrate metabolic process
**1442899_at**			0.044	1.33	---
**1444693_at**	Cacnb2	calcium channel, voltage-dependent, beta 2 subunit	0.007	1.33	---
**1419905_s_at**	Hpgd	hydroxyprostaglandin dehydrogenase 15 (NAD)	0.017	1.30	lipid metabolic process
**1421721_a_at**	Arnt	aryl hydrocarbon receptor nuclear translocator	0.015	1.28	response to hypoxia
**1424671_at**	Plekhf1	pleckstrin homology domain containing, family F (with FYVE domain) member 1	0.048	1.27	apoptotic process
**1417359_at**	Mfap2	microfibrillar-associated protein 2	0.034	1.27	platelet formation
**1422853_at**	Shc1	src homology 2 domain-containing transforming protein C1	0.025	1.26	MAPK cascade
**1460366_at**	Eml3	echinoderm microtubule associated protein like 3	0.026	1.24	proteolysis
**1460411_s_at**	AW548124	expressed sequence AW548124	0.044	1.23	ossification
**1456641_at**	1190007F08Rik	RIKEN cDNA 1190007F08 gene	0.035	1.23	---
**1453904_at**	4930528G09Rik	RIKEN cDNA 4930528G09 gene	0.045	1.22	---
**1422738_at**	Ddr2	discoidin domain receptor family, member 2	0.046	1.22	ossification
**1452289_a_at**	Rnf135	ring finger protein 135	0.011	1.22	protein ubiquitination
**1418394_a_at**	Cd97	CD97 antigen	0.016	1.22	cell adhesion
**1452527_a_at**	P2rx4	purinergic receptor P2X, ligand-gated ion channel 4	0.017	1.20	regulation of sodium ion transport
**AFFX-18SRNAMur/ X00686_M_at**			0.028	1.20	---
**1427547_a_at**	Slc26a3	solute carrier family 26, member 3	0.046	1.19	transport
**1431203_at**	Sdccag8	serologically defined colon cancer antigen 8	0.010	1.19	establishment of cell polarity
**1453826_at**	Pard3b	par-3 partitioning defective 3 homolog B (C. elegans)	0.005	1.19	cell cycle

**1417281_a_at**	Mmp23	matrix metallopeptidase 23	0.006	1.18	reproduction
**1422155_at**	Hist2h3c2	histone cluster 2, H3c2	0.039	1.17	negative regulation of transcription from RNA polymerase II promoter
**1437913_at**	Bcl2a1b	B-cell leukemia/lymphoma 2 related protein A1b	0.004	1.17	B cell homeostasis
**1458512_at**	Tle3	transducin-like enhancer of split 3, homolog of Drosophila E(spl)	0.048	1.17	transcription DNA-dependent
**1448756_at**	S100a9	S100 calcium binding protein A9 (calgranulin B)	0.040	1.17	chronic inflammatory response
**1424692_at**	2810055F11Rik	RIKEN cDNA 2810055F11 gene	0.047	1.16	---
**1418824_at**	Arf6	ADP-ribosylation factor 6	0.022	1.16	liver development
**1446313_at**	Zfp30	zinc finger protein 30	0.043	1.16	transcription DNA-dependent
**1425012_at**	Gng5	guanine nucleotide binding protein (G protein), gamma 5 subunit	0.012	1.15	signal transduction
**1430397_at**	Got2	glutamate oxaloacetate transaminase 2, mitochondrial	0.016	1.15	2-oxoglutarate metabolic process
**1447850_x_at**	Zfand3	zinc finger, AN1-type domain 3	0.045	1.15	---
**1451099_at**	Mbc2	membrane bound C2 domain containing protein	0.026	1.15	---
**1440649_at**	Tes	testis derived transcript	0.021	1.14	---
**1420801_at**	Npas1	neuronal PAS domain protein 1	0.030	1.14	negative regulation of transcription from RNA polymerase II promoter
**1441535_at**	Mllt3	myeloid/lymphoid or mixed lineage-leukemia translocation to 3 homolog (Drosophila)	0.018	1.14	---
**1441179_at**	9530020O07Rik	RIKEN cDNA 9530020O07 gene	0.029	1.14	---
**1448626_at**	Cdk5rap1	CDK5 regulatory subunit associated protein 1	0.015	1.14	regulation of cyclin-dependent protein kinase activity
**1422699_at**	Alox12	arachidonate 12-lipoxygenase	0.029	1.14	leukotriene metabolic process
**1428394_at**	Phyhd1	phytanoyl-CoA dioxygenase domain containing 1	0.021	1.14	pre-B cell differentiation
**1443029_at**	Ppfibp1	PTPRF interacting protein, binding protein 1 (liprin beta 1)	0.001	1.14	---
**1446869_at**			0.042	1.14	---
**1442724_at**	AW060763	expressed sequence AW060763	0.029	1.14	embryo development
**1432878_at**	4930544N03Rik	RIKEN cDNA 4930544N03 gene	0.012	1.14	---
**1453926_at**	Rad54l	RAD54 like (S. cerevisiae)	0.035	1.14	double-strand break repair via homologous recombination
**1435622_at**	Hs3st3a1	heparan sulfate (glucosamine) 3-O-sulfotransferase 3A1	0.012	1.13	---
**1453924_a_at**	Ptgfr	prostaglandin F receptor	0.047	1.13	signal transduction
**1417267_s_at**	Fkbp11	FK506 binding protein 11	0.004	1.13	protein peptidyl-prolyl isomerization
**1417860_a_at**	Spon2	spondin 2, extracellular matrix protein	0.017	1.13	cell adhesion

**1420195_at**			0.034	1.13	---
**1452264_at**	Tenc1	tensin like C1 domain-containing phosphatase	0.023	1.13	kidney development
**1416847_s_at**	Oas1d	2'-5' oligoadenylate synthetase 1D	0.021	1.13	ovarian follicle development
**1441885_s_at**			0.005	1.13	---
**1427880_at**	Uqcrq	ubiquinol-cytochrome c reductase, complex III subunit VII	0.029	1.13	transport
**1419541_at**	4930403L05Rik	RIKEN cDNA 4930403L05 gene	0.020	1.13	regulation of signal transduction
**1453295_at**	Asxl2	additional sex combs like 2 (Drosophila)	0.003	1.13	transcription DNA-dependent
**1436903_at**	Ubqln3	ubiquilin 3	0.020	1.13	---
**1443325_at**	Erh	enhancer of rudimentary homolog (Drosophila)	0.002	1.13	---
**1432139_at**	Srpk3	serine/arginine-rich protein specific kinase 3	0.032	1.13	protein phosphorylation
**1444324_at**	E530001K10Rik	RIKEN cDNA E530001K10 gene	0.044	1.13	---
**1420894_at**	Tgfbr1	transforming growth factor, beta receptor I	0.045	1.12	activation of MAPKK activity
**1440477_at**			0.012	1.12	---
**1441561_at**	Fbxl3	F-box and leucine-rich repeat protein 3	0.008	1.12	protein ubiquitination
**1421220_at**	Ankrd17	ankyrin repeat domain 17	0.014	1.12	endoderm development
**1458486_at**			0.030	1.12	---
**1430766_at**	5033403F01Rik	RIKEN cDNA 5033403F01 gene	0.008	1.12	---
**1424793_a_at**	Pbp2	phosphatidylethanolamine binding protein 2	0.002	1.12	negative regulation of peptidase activity
**1426197_at**	EG629860	predicted gene, EG629860	0.004	1.12	---
**1438097_at**	Rab20	RAB20, member RAS oncogene family	0.038	1.11	transport
**1447350_at**			0.033	1.11	---
**1454580_at**	5430427N15Rik	RIKEN cDNA 5430427N15 gene	0.040	1.11	---
**1450621_a_at**	Hbb-y	hemoglobin Y, beta-like embryonic chain	0.008	1.11	negative regulation of transcription from RNA polymerase II promoter
**1430747_at**	Pqlc1	PQ loop repeat containing 1	0.026	1.11	---
**1430321_at**	Krba1	KRAB-A domain containing 1	0.039	1.11	---
**1442673_at**	Stam2	signal transducing adaptor molecule (SH3 domain and ITAM motif) 2	0.037	1.11	---
**1438775_at**			0.035	1.11	---
**1424660_s_at**	Crtc2	CREB regulated transcription coactivator 2	0.011	1.11	gluconeogenesis
**1423541_at**	4930511I11Rik	RIKEN cDNA 4930511I11 gene	0.023	1.11	---
**1422231_a_at**	Tnfrsf25	tumor necrosis factor receptor superfamily, member 25	0.036	1.11	signal transduction
**1457509_at**	Sbno1	sno, strawberry notch homolog 1 (Drosophila)	0.026	1.10	---
**1430505_at**	1110032O16Rik	RIKEN cDNA 1110032O16 gene	0.017	1.10	---
**1459363_at**	Atxn2	ataxin 2	0.014	1.10	negative regulation of receptor internalization
**1443840_x_at**	1700010H22Rik	RIKEN cDNA 1700010H22 gene	0.025	1.10	---
**1448003_at**	1810043H04Rik	RIKEN cDNA 1810043H04 gene	0.007	1.10	---
**1429871_at**	Hmmr	hyaluronan mediated motility receptor	0.028	1.10	---

		(RHAMM)			
**1449178_at**	Pdlim3	PDZ and LIM domain 3	0.036	1.10	actin filament organization
**1445620_at**			0.015	1.10	---
**1441697_at**	Maml1	mastermind like 1 (Drosophila)	0.010	1.10	transcription DNA-dependent
**1449642_at**	Ebi2	Epstein-Barr virus induced gene 2	0.043	1.10	---
**1459322_at**	Syne2	synaptic nuclear envelope 2	0.039	1.10	---
**1446501_at**	A830053O21Rik	RIKEN cDNA A830053O21 gene	0.043	1.10	transcription DNA-dependent
**1441220_at**	Magi2	membrane associated guanylate kinase, WW and PDZ domain containing 2	0.049	1.10	---
**1432009_at**	4933415D12Rik	RIKEN cDNA 4933415D12 gene	0.047	1.10	---
**1421898_a_at**	Mr1	major histocompatibility complex, class I-related	0.028	1.10	antigen processing and presentation of peptide antigen via MHC class I
**1443084_at**	Ctso	cathepsin O	0.007	1.09	proteolysis
**1454326_at**	1700017H01Rik	RIKEN cDNA 1700017H01 gene	0.011	1.09	---
**1440782_at**	Skap1	src family associated phosphoprotein 1	0.044	1.09	positive regulation of transcription, DNA-dependent
**1439922_at**	Prrc1	proline-rich coiled-coil 1	0.023	1.09	---
**1445153_at**	Abl1	v-abl Abelson murine leukemia oncogene 1	0.029	1.09	---
**1425585_at**	Med12	mediator of RNA polymerase II transcription, subunit 12 homolog (yeast)	0.040	1.09	neural tube closure
**1458532_at**	Mtr	5-methyltetrahydrofolate-homocysteine methyltransferase	0.022	1.09	protein methylation
**1453305_at**	Iqcd	IQ motif containing D	0.023	1.09	---
**1439081_at**	Mgea5	meningioma expressed antigen 5 (hyaluronidase)	0.012	1.09	N-acetylglucosamine metabolic process
**1418578_at**	Dgka	diacylglycerol kinase, alpha	0.008	1.09	protein kinase C-activating G-protein coupled receptor signaling pathway
**1451443_at**	Nfix	nuclear factor I/X	0.036	1.09	negative regulation of transcription from RNA polymerase II promoter
**1437872_at**	AB112350	cDNA sequence AB112350	0.033	1.09	lipid metabolic process
**1429879_at**	0610037L13Rik	RIKEN cDNA 0610037L13 gene	0.008	1.09	---
**1439140_at**	1700019P01Rik	RIKEN cDNA 1700019P01 gene	0.017	1.09	---
**1430486_at**	Rad51l1	RAD51-like 1 (S. cerevisiae)	0.009	1.09	DNA metabolic process
**1448839_at**	Ankrd47	ankyrin repeat domain 47	0.041	1.09	negative regulation of stress fiber assembly
**1443468_at**			0.033	1.09	---
**1442547_at**			0.027	1.09	---
**1441366_at**			0.016	1.08	---
**1441568_at**	Osgin2	oxidative stress induced growth inhibitor family member 2	0.032	1.08	---
**1430916_at**	Fancd2	Fanconi anemia, complementation group D2	0.038	1.08	DNA repair
**1421443_at**	Gpr110	G protein-coupled receptor 110	0.018	1.08	signal transduction

**1428058_at**	Ahnak	AHNAK nucleoprotein (desmoyokin)	0.026	1.08	---
**1440677_at**	EG245305	predicted gene, EG245305	0.048	1.08	regulation of transcription, DNA-dependent
**1439376_x_at**			0.018	1.08	transcription DNA-dependent
**1440126_at**	BC037704	cDNA sequence BC037704	0.026	1.08	---
**1422565_s_at**	Nfic	nuclear factor I/C	0.047	1.08	negative regulation of transcription from RNA polymerase II promoter
**1443112_at**	Api5	apoptosis inhibitor 5	0.040	1.08	apoptotic process
**1448423_at**	Tmed4	transmembrane emp24 protein transport domain containing 4	0.029	1.08	transport
**1424304_at**	Tpcn2	two pore segment channel 2	0.012	1.08	transport
**1459520_at**	Gnb5	guanine nucleotide binding protein, beta 5	0.022	1.08	---
**1442496_at**	Cdkl3	cyclin-dependent kinase-like 3	0.023	1.08	---
**1459958_at**	Rsrc1	arginine/serine-rich coiled-coil 1	0.023	1.08	alternative nuclear mRNA splicing, via spliceosome
**1439970_at**	Wnk1	WNK lysine deficient protein kinase 1	0.033	1.07	---
**1431916_at**	Hsd3b3	hydroxy-delta-5-steroid dehydrogenase, 3 beta- and steroid delta-isomerase 3	0.024	1.07	steroid biosynthetic process
**1439110_at**	A930012O16Rik	RIKEN cDNA A930012O16 gene	0.034	1.07	---
**1429110_a_at**	Nsun4	NOL1/NOP2/Sun domain family, member 4	0.042	1.07	methylation
**1441089_at**	Eif2c1	eukaryotic translation initiation factor 2C, 1	0.047	1.07	mRNA catabolic process
**1441755_at**	Mapk15	mitogen-activated protein kinase 15	0.008	1.07	negative regulation of transcription from RNA polymerase II promoter
**1458258_at**			0.019	1.07	---
**1436631_at**	Ctnna1	catenin (cadherin associated protein), alpha 1	0.037	1.07	ovarian follicle development
**1453686_x_at**	Nphp1	nephronophthisis 1 (juvenile) homolog (human)	0.041	1.07	spermatogenesis
**1443917_at**	Tmbim4	transmembrane BAX inhibitor motif containing 4	0.024	1.07	apoptotic process
**1424787_a_at**	Nrf1	nuclear respiratory factor 1	0.037	1.07	response to hypoxia
**1456297_at**	Slc46a2	solute carrier family 46, member 2	0.045	1.07	transport
**1456808_at**	4933426M11Rik	RIKEN cDNA 4933426M11 gene	0.017	1.07	---
**1446450_at**	Ttn	titin	0.032	1.07	in utero embryonic development
**1438762_at**	Tcf12	transcription factor 12	0.048	1.06	---
**1450468_at**	Myoc	myocilin	0.014	1.06	---
**1425675_s_at**	Ceacam1	CEA-related cell adhesion molecule 1	0.027	1.06	signal transduction
**1444482_at**	A130078K24Rik	RIKEN cDNA A130078K24 gene	0.047	1.06	---
**1442578_at**			0.027	1.06	transcription DNA-dependent
**1440909_at**	Irgc1	immunity-related GTPase family, cinema 1	0.039	1.06	---
**1417605_s_at**	Camk1	calcium/calmodulin-dependent protein kinase I	0.048	1.06	protein phosphorylation
**1437840_s_at**			0.028	1.06	response to manganese ion
**1453969_at**	Prl8a1	prolactin family 8, subfamily a, member 1	0.007	1.06	---
**1459060_at**	Shcbp1	Shc SH2-domain binding protein 1	0.038	1.06	---

**1433001_at**	1700019L13Rik	RIKEN cDNA 1700019L13 gene	0.041	1.06	---
**1429199_s_at**	Saal1	serum amyloid A-like 1	0.037	1.06	acute-phase response
**1419936_at**	Csnk2a2	casein kinase 2, alpha prime polypeptide	0.046	1.06	---
**1438694_at**	Csn1s2a	casein alpha s2-like A	0.040	1.05	transport
**1441321_at**	LOC552911	hypothetical LOC552911	0.048	1.05	---
**1444394_at**	Acad9	acyl-Coenzyme A dehydrogenase family, member 9	0.045	1.05	metabolic process
**1416148_at**	Laptm4b	lysosomal-associated protein transmembrane 4B	0.049	1.05	transport
**1446879_at**	Grid2	glutamate receptor, ionotropic, delta 2	0.029	1.05	---
**1416456_a_at**	Chia	chitinase, acidic	0.029	1.05	polysaccharide catabolic process
**1443655_s_at**	Pik3c2a	phosphatidylinositol 3-kinase, C2 domain containing, alpha polypeptide	0.025	1.05	exocytosis
**1422228_at**	Wnt8a	wingless-related MMTV integration site 8A	0.018	1.05	negative regulation of transcription from RNA polymerase II promoter
**1438783_at**	AW742560	expressed sequence AW742560	0.026	1.05	---
**1451544_at**	Tapbpl	TAP binding protein-like	0.016	1.05	---
**1442846_at**	Pbx1	pre B-cell leukemia transcription factor 1	0.035	1.05	---
**1443475_at**	Hist1h3i	histone cluster 1, H3i	0.033	1.05	nucleosome assembly
**1457577_at**			0.038	1.05	---
**1422187_at**	Gabrg3	gamma-aminobutyric acid (GABA-A) receptor, subunit gamma 3	0.038	1.05	transport
**1441677_at**	Smc4	structural maintenance of chromosomes 4	0.040	1.04	---
**1441400_at**	1810029B16Rik	RIKEN cDNA 1810029B16 gene	0.041	1.04	---
**1421555_at**	Adrb3	adrenergic receptor, beta 3	0.043	1.04	diet induced thermogenesis
**1445446_at**			0.041	1.04	---
**1434584_a_at**	Pramel5	preferentially expressed antigen in melanoma like 5	0.031	1.03	---
**1441718_at**	Ubr5	ubiquitin protein ligase E3 component n-recognin 5	0.022	1.03	---
**1431627_at**	Kif27	kinesin family member 27	0.038	1.02	microtubule-based movement
**1432568_at**	Cby3	chibby homolog 3 (Drosophila)	0.042	1.02	---
**1443117_at**	Eya1	eyes absent 1 homolog (Drosophila)	0.033	1.01	establishment of mitotic spindle orientation
**1457413_at**	Ror1	receptor tyrosine kinase-like orphan receptor 1	0.039	1.00	---

Table S2. Transcripts downregulated in AR100 primary motor neurons

Probe Set ID	Gene Symbol	Gene Title	p-value	FC	GO biological process term
**1430328_at**	Polr3f	polymerase (RNA) III (DNA directed) polypeptide F	0.015	-1.28	transcription DNA-dependent
**1425446_at**			0.008	-1.26	---
**1454068_at**	4930470H14Rik	RIKEN cDNA 4930470H14 gene	0.024	-1.25	---
**1433363_at**	A930036I15Rik	RIKEN cDNA A930036I15 gene	0.023	-1.24	---
**1451513_x_at**	Serpina1b	serine (or cysteine) preptidase inhibitor, clade A, member 1b	0.025	-1.24	in utero embryonic development
**1444435_at**	Vps13b	vacuolar protein sorting 13B (yeast)	0.039	-1.23	transport
**1442147_at**	Phka2	phosphorylase kinase alpha 2	0.012	-1.22	---
**1443933_at**	Mtac2d1	membrane targeting (tandem) C2 domain containing 1	0.002	-1.22	---
**1451892_at**	Kl	klotho	0.01	-1.22	acute inflammatory response
**1430936_at**	Chmp7	CHMP family, member 7	0.012	-1.21	transport
**1442639_at**			0.028	-1.21	---
**1432662_at**	0610042E11Rik	RIKEN cDNA 0610042E11 gene	0.032	-1.21	---
**1421506_at**	Olfr78	olfactory receptor 78	0.037	-1.21	signal transduction
**1434007_at**	Gyltl1b	glycosyltransferase-like 1B	0.027	-1.21	---
**1444665_at**	4930402E16Rik	RIKEN cDNA 4930402E16 gene	0.046	-1.21	glycine catabolic process
**1432449_at**	4930564B18Rik	RIKEN cDNA 4930564B18 gene	0.026	-1.2	---
**1453509_at**	Lypd2	Ly6/Plaur domain containing 2	0.003	-1.2	---
**1439544_at**	Tbc1d5	TBC1 domain family, member 5	0.009	-1.2	regulation of Rab GTPase activity
**1427572_at**	2400001E08Rik	RIKEN cDNA 2400001E08 gene	0.008	-1.2	---
**1431021_at**	Cyb561d1	cytochrome b-561 domain containing 1	0.012	-1.19	transport
**1427115_at**	Myh3	myosin, heavy polypeptide 3, skeletal muscle, embryonic	0.032	-1.19	---
**1460067_at**	Ccr2	chemokine (C-C motif) receptor 2	0.038	-1.19	angiogenesis
**1455445_at**	Cbln3	cerebellin 3 precursor protein	0.021	-1.19	---
**1446218_at**	C230021G24Rik	RIKEN cDNA C230021G24 gene	0.04	-1.19	---
**1444749_at**			0.006	-1.19	---
**1457554_at**	Apob	apolipoprotein B	0.032	-1.18	in utero embryonic development
**1416977_at**	Stam2	signal transducing adaptor molecule (SH3 domain and ITAM motif) 2	0.015	-1.18	transport
**1420470_at**	Sult1c1	sulfotransferase family, cytosolic, 1C, member 1	0.024	-1.18	sulfur compound metabolic process
**1432252_a_at**	4933406K04Rik	RIKEN cDNA 4933406K04 gene	0.028	-1.18	---
**1442814_at**	Glcci1	glucocorticoid induced transcript 1	0.037	-1.18	---
**1432340_at**	1700121N20Rik	RIKEN cDNA 1700121N20 gene	0.025	-1.18	---
**1447102_at**	Ninj2	ninjurin 2	0.008	-1.18	---
**1458895_at**	Ard1b	ARD1 homolog B (S. cerevisiae)	0.025	-1.18	---
**1424445_at**	Tm4sf5	transmembrane 4 superfamily member 5	0.005	-1.18	---
**1421222_at**	Fip1l1	FIP1 like 1 (S. cerevisiae)	0.024	-1.18	mRNA polyadenylation
**1442140_at**	Tnn	tenascin N	0.048	-1.18	cell-matrix adhesion

**1419175_a_at**	Btn1a1	butyrophilin, subfamily 1, member A1	0.023	-1.18	negative regulation of cellular metabolic process
**1425145_at**	Il1rl1	interleukin 1 receptor-like 1	0.022	-1.17	negative regulation of T-helper 1 type immune response
**1456949_at**	Fchsd2	FCH and double SH3 domains 2	0.044	-1.17	---
**1425215_at**	Ffar2	free fatty acid receptor 2	0.041	-1.17	signal transduction
**1427214_at**	Agmat	agmatine ureohydrolase (agmatinase)	0.023	-1.17	spermidine biosynthetic process
**1454210_at**	4933428M09Rik	RIKEN cDNA 4933428M09 gene	0.037	-1.17	---
**1447065_at**	EG330070	predicted gene, EG330070	0.033	-1.17	---
**1446212_at**			0.034	-1.17	---
**1445905_at**	C79206	expressed sequence C79206	0.036	-1.17	---
**1432465_at**	4933414I06Rik	RIKEN cDNA 4933414I06 gene	0.049	-1.16	---
**1445889_at**	Sorl1	sortilin-related receptor, LDLR class A repeats-containing	0.018	-1.16	---
**1447455_at**			0.042	-1.16	---
**1429823_at**	Sgcg	sarcoglycan, gamma (dystrophin-associated glycoprotein)	0.041	-1.16	cytoskeleton organization
**1419982_s_at**			0.013	-1.16	---
**1443043_at**	Otop2	otopetrin 2	0.022	-1.16	---
**1450274_at**	Gprc2a-rs5	G protein-coupled receptor, family C, group 2, member A, related sequence 5	0.034	-1.16	---
**1439628_x_at**	Rab38	Rab38, member of RAS oncogene family	0.04	-1.16	transport
**1444759_at**	Prickle1	prickle like 1 (Drosophila)	0.04	-1.16	neural tube closure
**1444516_at**	Ppfibp2	protein tyrosine phosphatase, receptor-type, F interacting protein, binding protein 2	0.019	-1.16	---
**1432118_at**	2410012E07Rik	RIKEN cDNA 2410012E07 gene	0.026	-1.16	---
**1419621_at**	Ankrd2	ankyrin repeat domain 2 (stretch responsive muscle)	0.006	-1.16	---
**1446233_at**	Adarb2	adenosine deaminase, RNA-specific, B2	0.015	-1.16	---
**1450604_at**	Olfr140	olfactory receptor 140	0.01	-1.16	signal transduction
**1432239_at**	1700102J08Rik	RIKEN cDNA 1700102J08 gene	0.004	-1.16	---
**1430932_at**	Slc9a8	solute carrier family 9 (sodium/hydrogen exchanger), member 8	0.023	-1.16	transport
**1444424_at**	Sae1	SUMO1 activating enzyme subunit 1	0.047	-1.15	---
**1424401_at**	Aldh1l1	aldehyde dehydrogenase 1 family, member L1	0.048	-1.15	one-carbon metabolic process
**1439714_at**			0.005	-1.15	---
**1432983_at**	1700026H06Rik	RIKEN cDNA 1700026H06 gene	0.043	-1.15	---
**1458751_at**			0.011	-1.15	---
**1457224_at**	2610528E23Rik	RIKEN cDNA 2610528E23 gene	0.049	-1.15	---
**1448002_x_at**	2610001J05Rik	RIKEN cDNA 2610001J05 gene	0.025	-1.15	---
**1443092_at**	Mtmr3	myotubularin related protein 3	0.022	-1.15	protein dephosphorylation
**1440606_at**			0.006	-1.15	---
**1428685_at**	Syce1	synaptonemal complex central element protein 1	0.023	-1.15	cell cycle
**1432755_at**	2210409O19Rik	RIKEN cDNA 2210409O19 gene	0.021	-1.15	---

**1424592_a_at**	Dnase1	deoxyribonuclease I	0.026	-1.15	DNA catabolic process
**1446595_at**	Itsn2	intersectin 2	0.029	-1.15	endocytosis
**1420253_at**	D2Ertd63e	DNA segment, Chr 2, ERATO Doi 63, expressed	0.026	-1.15	---
**1416725_at**	Tcf4	transcription factor 4	0.029	-1.15	negative regulation of transcription from RNA polymerase II promoter
**1426139_a_at**	Ccrl1	chemokine (C-C motif) receptor-like 1	0.023	-1.15	signal transduction
**1448872_at**	Reg3g	regenerating islet-derived 3 gamma	0.045	-1.15	acute-phase response
**1431817_at**	Adh6-ps1	alcohol dehydrogenase 6 (class V), pseudogene 1	0.027	-1.15	---
**1441351_at**	Ankib1	ankyrin repeat and IBR domain containing 1	0.039	-1.14	---
**1442973_at**	C80865	expressed sequence C80865	0.018	-1.14	---
**1442719_at**	Prmt3	protein arginine N-methyltransferase 3	0.016	-1.14	protein methylation
**1458962_at**	Tmed5	transmembrane emp24 protein transport domain containing 5	0.019	-1.14	transport
**1459234_at**	Alox5ap	arachidonate 5-lipoxygenase activating protein	0.04	-1.14	---
**1420152_at**			0.031	-1.14	---
**1421684_at**	2310051M13Rik	RIKEN cDNA 2310051M13 gene	0.047	-1.14	proteolysis
**1424689_at**	Prss32	protease, serine, 32	0.025	-1.14	proteolysis
**1442391_at**	4930519H02Rik	RIKEN cDNA 4930519H02 gene	0.043	-1.14	---
**1446292_at**	Spata5	spermatogenesis associated 5	0.031	-1.14	---
**1459726_at**	Ptprn2	protein tyrosine phosphatase, receptor type, N polypeptide 2	0.042	-1.14	---
**1445052_at**	Pcdh9	protocadherin 9	0.044	-1.14	---
**1424627_at**	Cst12	cystatin 12	0.01	-1.14	negative regulation of peptidase activity
**1419627_s_at**	Clec4n	C-type lectin domain family 4, member n	0.038	-1.14	positive regulation of I-kappaB kinase
**1442765_at**			0.022	-1.13	---
**1423344_at**	Epor	erythropoietin receptor	0.013	-1.13	heart morphogenesis
**1419969_at**	C77370	expressed sequence C77370	0.033	-1.13	S phase of mitotic cell cycle
**1429010_at**	Snrp70	U1 small nuclear ribonucleoprotein polypeptide A	0.009	-1.13	nuclear mRNA splicing, via spliceosome
**1427891_at**	Gimap6	GTPase, IMAP family member 6	0.01	-1.13	---
**1446413_at**	4930555G07Rik	RIKEN cDNA 4930555G07 gene	0.028	-1.13	---
**1440393_at**	Odz2	odd Oz/ten-m homolog 2 (Drosophila)	0.016	-1.13	---
**1440617_at**	Cpa6	carboxypeptidase A6	0.024	-1.13	proteolysis
**1420360_at**	Dkk1	dickkopf homolog 1 (Xenopus laevis)	0.043	-1.13	negative regulation of transcription from RNA polymerase II promoter
**1437352_at**	Acox3	acyl-Coenzyme A oxidase 3, pristanoyl	0.012	-1.13	lipid metabolic process
**1457442_at**	AW125324	expressed sequence AW125324	0.029	-1.12	---
**1449409_at**	Sult1c2	sulfotransferase family, cytosolic, 1C, member 2	0.028	-1.12	sulfation
**1423147_at**	Mat1a	methionine adenosyltransferase I, alpha	0.036	-1.12	S-adenosylmethionine biosynthetic process
**1454083_at**	4930440C22Rik	RIKEN cDNA 4930440C22 gene	0.012	-1.12	---
**1439597_at**	4932417H02Rik	RIKEN cDNA 4932417H02 gene	0.001	-1.12	---
**1456498_at**	Itga4	integrin alpha 4	0.011	-1.12	blood vessel remodeling

**1443273_at**	Epha3	Eph receptor A3	0.007	-1.12	---
**1437551_at**	4930504E06Rik	RIKEN cDNA 4930504E06 gene	0.046	-1.12	---
**1460682_s_at**	Ceacam2	CEA-related cell adhesion molecule 2	0.039	-1.12	signal transduction
**1420234_at**			0.01	-1.12	---
**1430682_at**	4930555I21Rik	RIKEN cDNA 4930555I21 gene	0.014	-1.12	---
**1441189_at**			0.005	-1.12	---
**1451925_at**	Eda	ectodysplasin-A	0.006	-1.12	hair follicle development
**1455466_at**	Gpr133	G protein-coupled receptor 133	0.035	-1.12	cell surface receptor signaling pathway
**1456893_at**			0.038	-1.12	---
**1445054_at**	Foxn2	forkhead box N2	0.016	-1.12	---
**1426551_at**	Sidt1	SID1 transmembrane family, member 1	0.036	-1.12	RNA interference
**1460322_at**	Chst3	carbohydrate (chondroitin 6/keratan) sulfotransferase 3	0.029	-1.12	carbohydrate metabolic process
**1450996_at**	Fshb	follicle stimulating hormone beta	0.013	-1.12	transforming growth factor beta receptor signaling pathway
**1444335_at**	Edem2	ER degradation enhancer, mannosidase alpha-like 2	0.012	-1.12	---
**1453254_at**	1700034O15Rik	RIKEN cDNA 1700034O15 gene	0.025	-1.12	---
**1445486_at**	Lrrc49	leucine rich repeat containing 49	0.033	-1.12	---
**1444608_at**	Ankrd17	ankyrin repeat domain 17	0.048	-1.12	---
**1454081_at**			0.034	-1.12	---
**1456744_x_at**	Flcn	folliculin	0.013	-1.12	regulation of protein phosphorylation
**1441184_at**	Trpc4ap	transient receptor potential cation channel, subfamily C, member 4 associated protein	0.004	-1.12	ubiquitin-dependent protein catabolic process
**1431139_at**	Cdyl2	chromodomain protein, Y chromosome-like 2	0.016	-1.11	metabolic process
**1453990_at**	4930554G24Rik	RIKEN cDNA 4930554G24 gene	0.024	-1.11	---
**1452554_at**	EG667141	predicted gene, EG667141	0.025	-1.11	---
**1449860_at**	Higd1b	HIG1 domain family, member 1B	0.019	-1.11	---
**1441476_at**	Socs2	suppressor of cytokine signaling 2	0.048	-1.11	lactation
**1449559_at**	Msx2	homeo box, msh-like 2	0.042	-1.11	negative regulation of transcription from RNA polymerase II promoter
**1441126_at**	Farsb	phenylalanyl-tRNA synthetase, beta subunit	0.033	-1.11	---
**1454370_at**	4930557B21Rik	RIKEN cDNA 4930557B21 gene	0.03	-1.11	---
**1419220_at**	Xirp1	xin actin-binding repeat containing 1	0.047	-1.11	heart morphogenesis
**1450861_at**	Fancc	Fanconi anemia, complementation group C	0.043	-1.11	myeloid cell homeostasis
**1448827_s_at**	Myh6	myosin, heavy polypeptide 6, cardiac muscle, alpha	0.045	-1.11	response to reactive oxygen species
**1453349_at**	Casz1	castor homolog 1, zinc finger (Drosophila)	0.014	-1.11	transcription DNA-dependent
**1446732_at**			0.005	-1.11	---
**1450364_a_at**	Havcr1	hepatitis A virus cellular receptor 1	0.02	-1.11	phagocytosis, engulfment
**1432214_at**	1700017D01Rik	RIKEN cDNA 1700017D01 gene	0.016	-1.11	---
**1458228_at**			0.031	-1.11	---
**1448731_at**	Il10ra	interleukin 10 receptor, alpha	0.045	-1.1	cytokine-mediated signaling pathway

**1457657_at**			0.025	-1.1	---
**1429361_at**	Pmch	pro-melanin-concentrating hormone	0.033	-1.1	regulation of heart rate
**1425651_at**	Nhej1	nonhomologous end-joining factor 1	0.024	-1.1	DNA repair
**1430657_at**	1810034E14Rik	RIKEN cDNA 1810034E14 gene	0.049	-1.1	---
**1420571_at**	Prl6a1	prolactin family 6, subfamily a, member 1	0.026	-1.1	---
**1446083_at**	Hnrpd	heterogeneous nuclear ribonucleoprotein D	0.028	-1.1	---
**1432559_at**	2210409D07Rik	RIKEN cDNA 2210409D07 gene	0.028	-1.1	---
**1446691_at**	Pdgfd	platelet-derived growth factor, D polypeptide	0.027	-1.1	multicellular organismal development
**1457093_at**	Gapvd1	GTPase activating protein and VPS9 domains 1	0.044	-1.1	endocytosis
**1445663_at**			0.04	-1.1	---
**1445333_at**			0.038	-1.1	---
**1423397_at**	Ugt2b5	UDP glucuronosyltransferase 2 family, polypeptide B5	0.019	-1.1	metabolic process
**1436419_a_at**	1700097N02Rik	RIKEN cDNA 1700097N02 gene	0.028	-1.1	---
**1423178_at**	Abi1	abl-interactor 1	0.025	-1.1	somitogenesis
**1454219_at**	Dnajc2	DnaJ (Hsp40) homolog, subfamily C, member 2	0.013	-1.1	G2 phase of mitotic cell cycle
**1450548_at**	Htr1f	5-hydroxytryptamine (serotonin) receptor 1F	0.012	-1.1	signal transduction
**1417447_at**	Tcf21	transcription factor 21	0.021	-1.1	negative regulation of transcription from RNA polymerase II promoter
**1425450_at**	Chi3l4	chitinase 3-like 4	0.01	-1.1	carbohydrate metabolic process
**1458766_at**	Pak7	p21 (CDKN1A)-activated kinase 7	0.018	-1.1	---
**1433036_at**	4930564B12Rik	RIKEN cDNA 4930564B12 gene	0.039	-1.1	---
**1445951_at**	C86345	expressed sequence C86345	0.003	-1.1	---
**1457920_at**	C130026L21Rik	RIKEN cDNA C130026L21 gene	0.038	-1.1	---
**1427570_at**	Srpr	signal recognition particle receptor ('docking protein')	0.016	-1.1	GTP catabolic process
**1440072_at**	E430022K19Rik	RIKEN cDNA E430022K19 gene	0.023	-1.1	---
**1459760_at**	Ndufs4	NADH dehydrogenase (ubiquinone) Fe-S protein 4	0.019	-1.1	---
**1446277_at**			0.009	-1.1	---
**1432732_at**	5830437K03Rik	RIKEN cDNA 5830437K03 gene	0.049	-1.09	---
**1451661_at**	Akap4	A kinase (PRKA) anchor protein 4	0.031	-1.09	transmembrane receptor protein serine
**1458055_at**	Dsc3	desmocollin 3	0.014	-1.09	---
**1432226_at**	4930548G14Rik	RIKEN cDNA 4930548G14 gene	0.043	-1.09	---
**1452564_at**	Ofa	oncofetal antigen	0.043	-1.09	---
**1453268_at**	Thg1l	tRNA-histidine guanylyltransferase 1-like (S. cerevisiae)	0.018	-1.09	tRNA modification
**1445603_at**	Sri	sorcin	0.041	-1.09	---
**1459187_at**	St3gal4	ST3 beta-galactoside alpha-2,3-sialyltransferase 4	0.029	-1.09	---
**1427478_at**	Usp12	ubiquitin specific peptidase 12	0.021	-1.09	proteolysis
**1431470_a_at**	4933414I15Rik	RIKEN cDNA 4933414I15 gene	0.038	-1.09	---
**1420962_at**	Hapln2	hyaluronan and proteoglycan link protein 2	0.01	-1.09	cell adhesion
**1450229_at**	Med14	mediator complex subunit 14	0.033	-1.09	transcription DNA-dependent
**1441862_at**	Ppox	protoporphyrinogen oxidase	0.021	-1.09	porphyrin-containing compound

					biosynthetic process
**1445875_at**	A230057D06Rik	RIKEN cDNA A230057D06 gene	0.028	-1.09	---
**1433483_s_at**	C86187	expressed sequence C86187	0.047	-1.09	---
**1446051_at**	Gm1140	gene model 1140, (NCBI)	0.028	-1.09	---
**1458573_at**			0.023	-1.09	---
**1457846_at**	Cox11	COX11 homolog, cytochrome c oxidase assembly protein (yeast)	0.011	-1.09	---
**1458981_at**	Wdr70	WD repeat domain 70	0.033	-1.09	---
**1433039_at**	4930555B11Rik	RIKEN cDNA 4930555B11 gene	0.042	-1.09	---
**1446878_at**	Lonrf2	LON peptidase N-terminal domain and ring finger 2	0.005	-1.09	---
**1445294_at**			0.046	-1.09	---
**1459567_at**	C77905	expressed sequence C77905	0.025	-1.09	---
**1445780_at**			0.005	-1.09	---
**1418725_at**	Dnajc5b	DnaJ (Hsp40) homolog, subfamily C, member 5 beta	0.03	-1.09	protein folding
**1460472_at**	Cdk3	cyclin-dependent kinase 3	0.026	-1.09	protein phosphorylation
**1445007_at**	Lrrc50	leucine rich repeat containing 50	0.037	-1.09	---
**1458257_at**			0.036	-1.09	---
**1433006_at**	5430430B14Rik	RIKEN cDNA 5430430B14 gene	0.046	-1.09	---
**1453553_at**			0.033	-1.09	---
**1450609_at**	Lcn8	lipocalin 8	0.021	-1.09	transport
**1429807_at**	1700011H22Rik	RIKEN cDNA 1700011H22 gene	0.048	-1.09	---
**1445170_at**	2700012I20Rik	RIKEN cDNA 2700012I20 gene	0.025	-1.09	---
**1431186_at**	Dlg5	discs, large homolog 5 (Drosophila)	0.02	-1.08	regulation of apoptotic process
**1459089_at**	RP23-292J1.1	hypothetical LOC545637	0.027	-1.08	---
**1426182_a_at**	Klrc1	killer cell lectin-like receptor subfamily C, member 1	0.048	-1.08	---
**1453477_at**	Zc3h6	zinc finger CCCH type containing 6	0.049	-1.08	---
**1440528_at**			0.04	-1.08	---
**1419764_at**	Chi3l3	chitinase 3-like 3	0.044	-1.08	carbohydrate metabolic process
**1430096_at**	2900017F05Rik	RIKEN cDNA 2900017F05 gene	0.046	-1.08	---
**1419346_a_at**	Svs5	seminal vesicle secretory protein 5	0.022	-1.08	---
**1445877_at**	Rfesd	Rieske (Fe-S) domain containing	0.021	-1.08	oxidation-reduction process
**1441758_at**	Rhot1	ras homolog gene family, member T1	0.027	-1.08	GTP catabolic process
**1442629_at**	Nt5c2	5'-nucleotidase, cytosolic II	0.032	-1.08	---
**1431633_x_at**	4930526L06Rik	RIKEN cDNA 4930526L06 gene	0.025	-1.08	---
**1457222_at**	Creb5	cAMP responsive element binding protein 5	0.011	-1.08	transcription DNA-dependent
**1446438_at**	Ubr1	ubiquitin protein ligase E3 component n-recognin 1	0.027	-1.08	ubiquitin-dependent protein catabolic process
**1458611_at**			0.031	-1.08	---
**1450774_at**	Ly6g6d	lymphocyte antigen 6 complex, locus G6D	0.041	-1.08	---
**1432623_at**	Supv3l1	suppressor of var1, 3-like 1 (S. cerevisiae)	0.034	-1.08	mitochondrial mRNA catabolic process
**1453489_at**	1700020O03Rik	RIKEN cDNA 1700020O03 gene	0.047	-1.08	---

**1445439_at**	Epb4.9	erythrocyte protein band 4.9	0.047	-1.08	cytoskeleton organization
**1459732_at**			0.038	-1.08	---
**1444332_at**	9130019P16Rik	RIKEN cDNA 9130019P16 gene	0.022	-1.08	---
**1440778_x_at**	Zfp712	zinc finger protein 712	0.041	-1.08	regulation of transcription, DNA-dependent
**1427818_at**	Bcl2	B-cell leukemia/lymphoma 2	0.028	-1.08	---
**1421637_at**	Slc5a4a	solute carrier family 5, member 4a	0.046	-1.08	transport
**1432647_at**	Egfr	epidermal growth factor receptor	0.044	-1.08	activation of MAPKK activity
**1437721_at**	Coro1c	coronin, actin binding protein 1C	0.031	-1.08	actin cortical patch assembly
**1443994_at**	6030407O03Rik	RIKEN cDNA 6030407O03 gene	0.047	-1.07	---
**1454970_at**	Ptcd1	pentatricopeptide repeat domain 1	0.025	-1.07	---
**1446541_at**	4930434E21Rik	RIKEN cDNA 4930434E21 gene	0.022	-1.07	---
**1454417_at**	4933408M05Rik	RIKEN cDNA 4933408M05 gene	0.032	-1.07	---
**1453827_at**	1110035H17Rik	RIKEN cDNA 1110035H17 gene	0.04	-1.07	---
**1441524_at**	Tec	cytoplasmic tyrosine kinase, Dscr28C related (Drosophila)	0.048	-1.07	---
**1422707_at**	Pik3cg	phosphoinositide-3-kinase, catalytic, gamma polypeptide	0.042	-1.07	positive regulation of acute inflammatory response
**1447104_at**	Arid1a	AT rich interactive domain 1A (Swi1 like)	0.031	-1.07	---
**1433370_at**	4930474A20Rik	RIKEN cDNA 4930474A20 gene	0.04	-1.07	---
**1451781_at**	Nfatc2ip	nuclear factor of activated T-cells, cytoplasmic, calcineurin-dependent 2 interacting protein	0.018	-1.07	cytokine production
**1442485_at**	9030205A07Rik	RIKEN cDNA 9030205A07 Gene	0.029	-1.07	---
**1449782_at**	AA517650	expressed sequence AA517650	0.014	-1.07	---
**1432560_at**	1700127D06Rik	RIKEN cDNA 1700127D06 gene	0.025	-1.07	proteolysis
**1455661_at**	ENSMUSG00000054945	predicted gene, ENSMUSG00000054945	0.043	-1.07	---
**1459597_at**	Mtpn	myotrophin	0.033	-1.07	catecholamine metabolic process
**1453585_at**	1600010M07Rik	RIKEN cDNA 1600010M07 gene	0.008	-1.07	---
**1449801_at**			0.043	-1.07	---
**1452405_x_at**	A430107P09Rik	RIKEN cDNA A430107P09 gene	0.015	-1.06	---
**1449963_at**	2310040M23Rik	RIKEN cDNA 2310040M23 gene	0.016	-1.06	---
**1458288_at**	4933426I21Rik	RIKEN cDNA 4933426I21 gene	0.031	-1.06	---
**1422933_at**	Xlr5c	X-linked lymphocyte-regulated 5C	0.049	-1.06	---
**1420293_at**	Aytl2	acyltransferase like 2	0.03	-1.06	phospholipid metabolic process
**1457739_at**	Ilf3	interleukin enhancer binding factor 3	0.022	-1.06	transcription DNA-dependent
**1438644_x_at**	Commd9	COMM domain containing 9	0.035	-1.06	---
**1427779_a_at**	Cd4	CD4 antigen	0.034	-1.06	cytokine production
**1437089_at**	4833409A17Rik	RIKEN cDNA 4833409A17 gene	0.012	-1.06	oxidation-reduction process
**1457391_at**	Vamp3	vesicle-associated membrane protein 3	0.016	-1.06	positive regulation of receptor recycling
**1460073_at**	Prkar1b	protein kinase, cAMP dependent regulatory, type I beta	0.033	-1.06	---
**1425370_a_at**	Erg	avian erythroblastosis virus E-26 (v-ets) oncogene related	0.044	-1.06	transcription DNA-dependent

**1421564_at**	Serpina3c	serine (or cysteine) peptidase inhibitor, clade A, member 3C	0.026	-1.06	negative regulation of peptidase activity
**1421185_at**	Mettl7a	methyltransferase like 7A	0.02	-1.06	metabolic process
**1441176_at**			0.042	-1.06	---
**1420108_at**			0.043	-1.06	---
**1444173_at**			0.038	-1.05	---
**1420379_at**	Slco1a1	solute carrier organic anion transporter family, member 1a1	0.039	-1.05	transport
**1445957_at**	Evl	Ena-vasodilator stimulated phosphoprotein	0.025	-1.05	---
**1441106_at**	Zfand5	zinc finger, AN1-type domain 5	0.009	-1.05	in utero embryonic development
**1428915_at**	Sirt5	sirtuin 5 (silent mating type information regulation 2 homolog) 5 (S. cerevisiae)	0.04	-1.05	protein deacetylation
**1436615_a_at**	Otc	ornithine transcarbamylase	0.036	-1.05	urea cycle
**1444888_at**	AU022852	expressed sequence AU022852	0.026	-1.05	---
**1449608_a_at**			0.032	-1.05	---
**1432773_at**	2610105M22Rik	RIKEN cDNA 2610105M22 gene	0.025	-1.05	---
**1443351_at**	Glra2	glycine receptor, alpha 2 subunit	0.032	-1.05	---
**1450578_at**	Sry	sex determining region of Chr Y	0.037	-1.04	transcription, DNA-dependent
**1458380_at**	Asxl1	additional sex combs like 1 (Drosophila)	0.043	-1.04	negative regulation of transcription from RNA polymerase II promoter
**1434068_s_at**	AI662270	expressed sequence AI662270	0.032	-1.04	---
**1449292_at**	Rb1cc1	RB1-inducible coiled-coil 1	0.049	-1.04	autophagic vacuole assembly
**1449475_at**	Atp12a	ATPase, H+/K+ transporting, nongastric, alpha polypeptide	0.042	-1.04	ATP biosynthetic process
**1435962_at**	Rps6	ribosomal protein S6	0.036	-1.03	ribosomal small subunit assembly
**1427614_at**	Pip	prolactin induced protein	0.048	-1.03	---
**1444115_at**			0.04	-1.03	---
**1453275_at**	2310002L13Rik	RIKEN cDNA 2310002L13 gene	0.038	-1.03	---
**1432690_at**	9030407C09Rik	RIKEN cDNA 9030407C09 gene	0.035	-1.03	---
**1444382_at**	4930579G22Rik	RIKEN cDNA 4930579G22 gene	0.045	-1.03	---
**1454433_at**	6330526H18Rik	RIKEN cDNA 6330526H18 gene	0.039	-1.03	---
**1417812_a_at**	Lamb3	laminin, beta 3	0.047	-1.03	cell adhesion
**1454216_at**	4930408F14Rik	RIKEN cDNA 4930408F14 gene	0.032	-1.02	gamete generation
**1444356_at**	Erc1	ELKS/RAB6-interacting/CAST family member 1	0.036	-1.02	---

Table S3. Identification of over-represented transcription factor binding sites (TFBS) a using oPOSSUM v3 software

TF	Class	Family	Target gene hits	Target TFBS hits	Z-score	Fisher score
**TBP**	Beta-sheet	TATA-binding	182	624	12.828	9.602
**PLAG1**	Zinc-coordinating	BetaBetaAlpha-zinc finger	38	49	11.289	9.949
**Foxa2**	Winged Helix-Turn-Helix	Forkhead	196	775	8.741	7.903
**Sox5**	Other Alpha-Helix	High Mobility Group	219	1231	8.534	8.633
**Evi1**	Zinc-coordinating	BetaBetaAlpha-zinc finger	43	52	8.448	9.692
**SOX9**	Other Alpha-Helix	High Mobility Group	206	781	8.043	12.714
**SRY**	Other Alpha-Helix	High Mobility Group	248	1722	7.799	11.36
**SRF**	Other Alpha-Helix	MADS	26	26	7.631	9.825
**HOXA5**	Helix-Turn-Helix	Homeo	277	3370	7.52	11.997
**Nkx2-5**	Helix-Turn-Helix	Homeo	267	3208	7.432	8.658
**Pdx1**	Helix-Turn-Helix	Homeo	244	2227	6.709	6.404
**ARID3A**	Helix-Turn-Helix	Arid	249	2607	6.643	4.899
**FOXD1**	Winged Helix-Turn-Helix	Forkhead	206	1098	6.494	4.186
**NR2F1**	Zinc-coordinating	Hormone-nuclear Receptor	75	109	6.039	8.762
**NFIL3**	Zipper-Type	Leucine Zipper	112	260	5.915	6.119
**IRF1**	Winged Helix-Turn-Helix	IRF	93	211	5.864	2.941
**NFATC2**	Ig-fold	Rel	233	1646	5.136	5.502
**INSM1**	Zinc-coordinating	BetaBetaAlpha-zinc finger	120	285	5.1	8.237

**Sox17**	Other Alpha-Helix	High Mobility Group	228	1185	4.869	8.993
**FOXO3**	Winged Helix-Turn-Helix	Forkhead	217	1212	4.804	5.095
**Foxd3**	Winged Helix-Turn-Helix	Forkhead	196	759	4.383	9.695
**FOXI1**	Winged Helix-Turn-Helix	Forkhead	207	812	4.296	11.43
**Foxq1**	Winged Helix-Turn-Helix	Forkhead	130	349	4.167	5.304
**NR3C1**	Zinc-coordinating	Hormone-nuclear Receptor	43	59	4.068	4.439
**HNF1A**	Helix-Turn-Helix	Homeo	55	71	4.011	8.206
**Stat3**	Ig-fold	Stat	167	431	3.928	10.858
**Prrx2**	Helix-Turn-Helix	Homeo	241	1863	3.889	8.937
**HNF1B**	Helix-Turn-Helix	Homeo	76	116	3.765	8.685
**SPI1**	Winged Helix-Turn-Helix	Ets	249	1746	3.602	9.943
**Nobox**	Helix-Turn-Helix	Homeo	210	1225	3.52	4.672
**NKX3-1**	Helix-Turn-Helix	Homeo	218	1155	3.49	8.181
**CREB1**	Zipper-Type	Leucine Zipper	161	373	3.076	11.689
**SP1**	Zinc-coordinating	BetaBetaAlpha-zinc finger	192	1169	3.015	9.049
**MEF2A**	Other Alpha-Helix	MADS	126	242	2.856	12.871
**Arnt::Ahr**	Zipper-Type	Helix-Loop-Helix	227	1317	2.581	10.868
**T**	Beta-Hairpin-Ribbon	T	35	44	2.393	4.303
**Pou5f1**	Helix-Turn-Helix	Homeo	57	79	2.377	6.394
**CEBPA**	Zipper-Type	Leucine Zipper	220	870	2.265	15.286

**PBX1**	Helix-Turn-Helix	Homeo	74	110	2.232	7.219
**FEV**	Winged Helix-Turn-Helix	Ets	234	1416	2.041	6.592
**Gata1**	Zinc-coordinating	GATA	229	1327	2.007	6.35
**CTCF**	Zinc-coordinating	BetaBetaAlpha-zinc finger	53	66	1.955	5.538
**YY1**	Zinc-coordinating	BetaBetaAlpha-zinc finger	267	3003	1.834	8.352
**MZF1_5-13**	Zinc-coordinating	BetaBetaAlpha-zinc finger	217	1272	1.807	10.471
**ELF5**	Winged Helix-Turn-Helix	Ets	268	2043	1.482	16.25
**PPARG::RXRA**	Zinc-coordinating	Hormone-nuclear Receptor	102	195	1.345	6.214
**Sox2**	Other Alpha-Helix	High Mobility Group	47	62	1.313	4.769
**FOXA1**	Winged Helix-Turn-Helix	Forkhead	213	1065	1.073	4.919
**NFKB1**	Ig-fold	Rel	54	92	0.954	3.123
**MIZF**	Zinc-coordinating	BetaBetaAlpha-zinc finger	41	45	0.939	6.196
**RREB1**	Zinc-coordinating	BetaBetaAlpha-zinc finger	28	33	0.858	3.727
**FOXF2**	Winged Helix-Turn-Helix	Forkhead	69	102	0.822	5.467
**Myb**	Helix-Turn-Helix	Myb	204	1034	0.549	4.958
**Tal1::Gata1**	Zipper-Type	Helix-Loop-Helix	102	192	0.487	4.4
**Pax6**	Helix-Turn-Helix	Homeo	23	24	0.448	3.606
**SPIB**	Winged Helix-Turn-Helix	Ets	277	3020	0.331	14.922
**Gfi**	Zinc-coordinating	BetaBetaAlpha-zinc finger	217	1049	0.165	7.114
**HNF4A**	Zinc-coordinating	Hormone-nuclear Receptor	113	205	0.056	7.215
**Ar**	Zinc-coordinating	Hormone-nuclear Receptor	9	10	-0.1	1.516

**Nkx3-2**	Helix-Turn-Helix	Homeo	246	1653	-0.184	9.21
**ELK4**	Winged Helix-Turn-Helix	Ets	100	170	-0.187	5.87
**NFYA**	Other Alpha-Helix	NFY CCAAT-binding	98	163	-0.213	6.992
**ESR1**	Zinc-coordinating	Hormone-nuclear Receptor	6	6	-0.292	1.427
**Nr2e3**	Zinc-coordinating	Hormone-nuclear Receptor	113	217	-0.508	6.129
**STAT1**	Ig-fold	Stat	106	177	-0.517	6.67
**GABPA**	Winged Helix-Turn-Helix	Ets	151	351	-0.714	8.983
**ZNF354C**	Zinc-coordinating	BetaBetaAlpha-zinc finger	267	3682	-0.758	9.107
**USF1**	Zipper-Type	Helix-Loop-Helix	149	391	-0.94	5.534
**MYC::MAX**	Zipper-Type	Helix-Loop-Helix	58	79	-1.112	4.118
**Ddit3::Cebpa**	Zipper-Type	Leucine Zipper	94	155	-1.271	5.781
**PPARG**	Zinc-coordinating	Hormone-nuclear Receptor	1	1	-1.337	0.512
**NHLH1**	Zipper-Type	Helix-Loop-Helix	88	149	-1.374	5.078
**RXRA::VDR**	Zinc-coordinating	Hormone-nuclear Receptor	8	8	-1.383	1.327
**EWSR1-FLI1**	Winged Helix-Turn-Helix	Ets	3	3	-1.487	0.788
**Lhx3**	Helix-Turn-Helix	Homeo	91	169	-1.5	5.325
**AP1**	Zipper-Type	Leucine Zipper	255	1992	-1.517	8.154
**IRF2**	Winged Helix-Turn-Helix	IRF	11	13	-1.521	1.018
**Spz1**	Other	Other	98	221	-1.639	1.563
**HLF**	Zipper-Type	Leucine Zipper	87	146	-1.679	4.32
**MZF1_1-4**	Zinc-coordinating	BetaBetaAlpha-zinc finger	260	3158	-1.864	10.733

**RUNX1**	Ig-fold	Runt	217	1095	-1.977	5.351
**E2F1**	Winged Helix-Turn-Helix	E2F	115	273	-2.009	3.591
**TEAD1**	Helix-Turn-Helix	Homeo	84	136	-2.041	4.236
**Pax5**	Helix-Turn-Helix	Homeo	18	22	-2.12	1.306
**Myc**	Zipper-Type	Helix-Loop-Helix	131	304	-2.146	6.194
**TLX1::NFIC**	Helix-Turn-Helix::Other	Homeo::Nuclear Factor I-CCAAT-binding	16	17	-2.181	1.652
**TP53**	Zinc-coordinating	Loop-Sheet-Helix	0	0	-2.268	0
**Myf**	Zipper-Type	Helix-Loop-Helix	148	455	-2.332	2.935
**NR4A2**	Zinc-coordinating	Hormone-nuclear Receptor	232	1112	-2.423	11.57
**TAL1::TCF3**	Zipper-Type	Helix-Loop-Helix	109	222	-2.624	2.972
**Tcfcp2l1**	Other	CP2	164	540	-2.636	5.136
**RELA**	Ig-fold	Rel	105	209	-2.639	3.448
**znf143**	Zinc-coordinating	BetaBetaAlpha-zinc finger	18	24	-2.677	0.796
**ELK1**	Winged Helix-Turn-Helix	Ets	198	927	-2.707	3.994
**Hand1::Tcfe2a**	Zipper-Type	Helix-Loop-Helix	206	964	-2.722	4.279
**Klf4**	Zinc-coordinating	BetaBetaAlpha-zinc finger	219	1359	-2.746	12.936
**Arnt**	Zipper-Type	Helix-Loop-Helix	105	222	-2.827	3.718
**MAX**	Zipper-Type	Helix-Loop-Helix	128	266	-3.016	5.247
**NR1H2::RXRA**	Zinc-coordinating	Hormone-nuclear Receptor	1	1	-3.021	0.294
**ESR2**	Zinc-coordinating	Hormone-nuclear Receptor	37	45	-3.658	2.18
**RORA_2**	Zinc-coordinating	Hormone-nuclear Receptor	56	74	-4.052	2.998
**NF-kappaB**	Ig-fold	Rel	121	284	-4.152	3.347

**EBF1**	Zipper-Type	Helix-Loop-Helix	166	633	-4.506	2.836
**Mycn**	Zipper-Type	Helix-Loop-Helix	126	293	-4.645	4.532
**REST**	Zinc-coordinating	BetaBetaAlpha-zinc finger	2	2	-4.774	0.289
**Esrrb**	Zinc-coordinating	Hormone-nuclear Receptor	170	458	-4.829	6.914
**HIF1A::ARNT**	Zipper-Type	Helix-Loop-Helix	161	604	-4.948	4.492
**REL**	Ig-fold	Rel	159	456	-5.188	4.661
**ZEB1**	Zinc-coordinating	BetaBetaAlpha-zinc finger	264	2890	-5.389	9.948
**RORA_1**	Zinc-coordinating	Hormone-nuclear Receptor	135	310	-5.722	3.039
**RXR::RAR_DR5**	Zinc-coordinating	Hormone-nuclear Receptor	19	20	-5.749	1.078
**NFE2L2**	Zipper-Type	Leucine Zipper	87	141	-5.855	2.757
**Zfp423**	Zinc-coordinating	BetaBetaAlpha-zinc finger	81	150	-5.89	2.23
**Egr1**	Zinc-coordinating	BetaBetaAlpha-zinc finger	82	158	-6.43	1.969
**Pax4**	Helix-Turn-Helix	Homeo	0	0	-6.728	0
**Zfx**	Zinc-coordinating	BetaBetaAlpha-zinc finger	149	427	-7.532	8.335

Table S4. qPCR primers

Primer	Primer Sequence
Actb F	ACAACGGCTCCGGCATGTGCAAAG
Actb R	CATTCCCACCATCACACCCTGGTGC
Ampkb1 F	TCCGAAGAGATCAAGGCTCC
Ampkb1 R	GACTTCCTTTCCACCCCCTG
Atg5 F	TTTGGGCCATCAACCGGAAA
Atg5 R	CGCTCCGTCGTGGTCTGATA
Atg7 F	AGCGGCGACAGCATTAGGAT
Atg7 R	CCTCATGGCAGGAAAGCAGT
Atg12 F	CCTTAAACTGGTGGCCTCGG
Atg12 R	ACCATCACTGCCAAAACACTCA
Cox1 F	GGTCAACCAGGTGCACTTTT
Cox1 R	TGGGGCTCCGATTATTAGTG
Cox2 F	TGAAGACGTCCTCCACTCATGA
Cox2 R	GCCTGGGATGGCATCAGTT
Cox3 F	GCAGGATTCTTCTGAGCGTTCT
Cox3 R	GTCAGCAGCCTCCTAGATCATGT
Dram1 F	CTGTCGTCCCCATGATTGCC
Dram1 R	CAAAAGCCACGGTCCACTCA
Gadd45 F	TGGTGACGAACCCACATTCA
Gadd45 R	CGGGAGATTAATCACGGGCA
Ho-1 F	GGTCAGGTGTCCAGAGAAGG
Ho-1 R	CTTCCAGGGCCGTGTAGATA
Hprt1 F	GCGTTGGGCTTACCTCACTGCT
Hprt1 R	ATCGCTAATCACGACGCTGGG
Igfbp3 F	TAAGAAGAAGCAGTGCCGCC
Igfbp3 R	TTTCCCCTTGGTGTCGTAGC
Lamp 1 F	CAGTTTGGGATGAATGCCAGC
Lamp 1 R	TGGAGATGCTGAATGTGGGC
Lamp 2 F	CAAAAGGACAGTATTCTACAGCTCA
Lamp 2 R	TGATGGCGCTTGAGACCAAT
Map1lc3b F	TAATCAGACGGCGCTTGCAG
Map1lc3b R	CTCACCATGCTGTGCCCATT
Nd1 F	CCTTCGACCTGACCTGACAGAAGGA
Nd1 R	GATGCTCGGATCCATAGGAA
Nd5 F	GCTCTACCTCACCATCTCTTGC
Nd5 R	TCCAGTATGCTTACCTTGTTACG
Nqo1 F	TTCTCTGGCCGATTCAGAGT
Nqo1 R	GGCTGCTTGGAGCAAAATG
Nrf2 F	TTCCTCTGCTGCCATTAGTCAGTC
Nrf2 R	GCTCTTCCATTTCCGAGTCACTG
Pgc1a F	TTGCTAGCGGTCCTCACAGA
Pgc1a R	GGCTCTTCTGCCTCCTGA
Pgc1b F	CGCTCCAGGAGACTGAATCCAG
Pgc1b R	CTTGACTACTGTCTGTGAGGC
Pgk1 F	TCTTGGGAGGCGCTAAAGTTG
Pgk1 R	AAGGCCATTCCACCACCAAT

Pparg F	GGAAGACCACTCGCATTCCTT
Pparg R	TCGCACTTTGGTATTCTTGGAG
Pten F	GCGGAACTTGCAATCCTCAG
Pten R	ACATGAACTTGTCCTCCCGC
Sesn1 F	GACGGACTTTCAAACGCGG
Sesn1 R	TTCCAAGTTCCTCGACGCTC
Sesn2 F	CGCCACTCAGAGAAGGTTCA
Sesn2 R	ACGGGGTAGTCAGGTCATGT
Sqstm1/p62 F	GCCAGAGGAACAGATGGAGTC
Sqstm1/p62 R	AGCTTGGCCCTTCCGATTC
Tfam F	CCAAAAAGACCTCGTTCAGC
Tfam R	ATGTCTCCGGATCGTTTCAC
Tsc2 F	ACCATCAGGTCCGAAAGCTG
Tsc2 R	CGCGCCATCACCTTTTCAAT

Table S5. TaqMan qPCR primers

TaqMan primer	Primer Code
Actin beta	Mm01205647_g1
Arnt	Mm00507836_m1
Chmp1a	Mm00848170_s1
Chmp1b	Mm00481091_s1
Chmp2a	Mm01187331_g1
Chmp2b	Mm00660653_m1
Chmp3	Mm01190252_m1
Chmp4b	Mm00551493_m1
Chmp4c	Mm00481500_m1
Chmp5	Mm00472587_m1
Chmp6	Mm01241413_g1
Chmp7	Mm00513649_m1
Dkk1	Mm00438422_m1
Dkk2	Mm01322146_m1
Dkk3	Mm00443800_m1
Hprt1	Mm01324427_m1
Itih5	Mm00552678_m1
Pgk1	Mm00435617_m1
Serping1	Mm00437834_m1
Actin beta	Hs99999903_m1
Chmp7	Hs01061798_m1
Pgk1	Hs99999906_m1

Supplementary Figure Legends

Fig. S1. Differential gene expression from spinal and bulbar muscular atrophy and wild-type mice is specific to embryonic motor neurons.

(A) TaqMan qPCR was performed to determine mRNA expression of indicated genes using the "ddCT" method of relative comparison on WT, AR20 and AR100 motor neurons. *Itih5, Serping 1*, *Arnt* and *Chmp7* were dysregulated only in AR100 and not AR20 motor neurons treated with DHT, relative to WT (B) The values obtained using qPCR correlated with microarray data. (C) The differential expression of these genes in AR100 motor neurons was further affected by DHT. (D) qPCR was performed to determine mRNA expression of genes in WT and AR100 cells cultured from meninges removed from embryonic spinal cords (predominantly fibroblasts), with and without DHT treatment. The expression of genes is not altered in these cultures (F) Astroglial cultures did not demonstrate appreciable changes in mRNA expression and any changes in gene expression of *Serping 1* and *Arnt* were in the opposite direction to motor neurons. Data are displayed as mean ± SEM and are representative of independent experiments from three replicate cultures. Statistical analysis was performed using a one-way ANOVA followed by the Student–Newman–Keuls and Tukey's Honestly Significantly Different post hoc tests (n≥3, $*P<0.05$).

Fig. S2. Gene ontology analysis of differentially expressed genes.

GO Slim analysis was performed on differentially expressed genes found to be altered in primary embryonic motor neurons from SBMA AR100 mice. In the Go slim biological process categories, significant genes were annotated to the signal transduction, anatomical structure development, transport, cell differentiation, cellular protein modification process, response to stress and cell death terms. In the molecular function categories, genes were mapped to the following terms: ion binding, signal transducer activity, DNA binding, enzyme binding, RNA binding and cytoskeletal protein binding

Fig. S3. ClueGo protein-protein interaction network and CluePedia enrichment

ClueGO analyses functional interaction of gene using GO information and KEGG, BioCarta and Reactome gene functional databases. A two-sided hypergeometric statistic was performed with kappa score threshold setting of 0.3. The kappa score measures the association strength between overlapping GO terms. Enrichment/depletion was calculated based on p-value corrected using the FDR method of Benjamini and Hochberg. CluePedia extends ClueGO functionality down to genes and miRNAs.

Fig. S4. Gene set enrichment analysis and String protein-protein network generation.

(A-B) GSEA analysis of gene expression data reveals upregulation of KEGG p53 pathway and enrichment of genes present in this pathway in AR100 motor neurons (shown in red) which were mapped using KEGG Mapper (https://www.genome.jp/kegg/mapper.html) and (B) enrichment of genes (in red) in KEGG Lysosome pathway.

Fig. S5. *Chmp7* expression in models of motor neuron disease

(A) qPCR was performed to determine the expression of *Chmp7* in the cortex of presymptomatic 3 month old AR100 and WT mice. (B) Spinal cord from presymptomatic 30 day old SOD1-G93A ALS mice was used to examine *Chmp7* expression**.** (C) qPCR was also used to examine expression of *Chmp7* in the spinal cord of presymptomatic 3 month old ALS4 SETX-R2136H mice. qPCR data are displayed as mean ± SEM and are representative of three independent experiments. Statistical analysis was performed using a two sample t-test (n=3, **P*<0.05).

Fig. S6. *Lc3b* is upregulated in the spinal cord of SBMA mice

(A) Genes associated with autophagy and the lysosome were examined using spinal cord from AR20 and AR100 presymptomatic mice of 3 months of age. Autophagy genes (*Atg5,*

Atg7, Atg12, *Lc3b and p62*) were not altered in AR100 mice. (B) In spinal cord of 12 month old symptomatic AR100 mice, there was an increase in *Lc3b* expression. (C) LAMP2 and β-III tubulin staining of AR100 and AR20 spinal cord motor neurons. qPCR data are displayed as mean ± SEM and are representative of three independent experiments. Statistical analysis was performed using a two sample t-test (n≥3, **P*<0.05)

Fig. S7. Uncropped Western blot images.

Uncropped Western blot images are displayed from Fig. 2F, G and I.

Fig. S8. Aberrant CHMP7 localisation in AR100 SBMA mice

Ventral horn spinal cord was immunostained with β-III tubulin and CHMP7. In presymptomatic 3 month old AR100 mice CHMP7 expression appeared reduced within motor neurons and was localised around the perinuclear region, while in WT mice CHMP7 displayed a more uniform pattern of staining within the cytoplasm and nucleus of motor neurons. Scale bars represent 20 μm.

Fig. S1

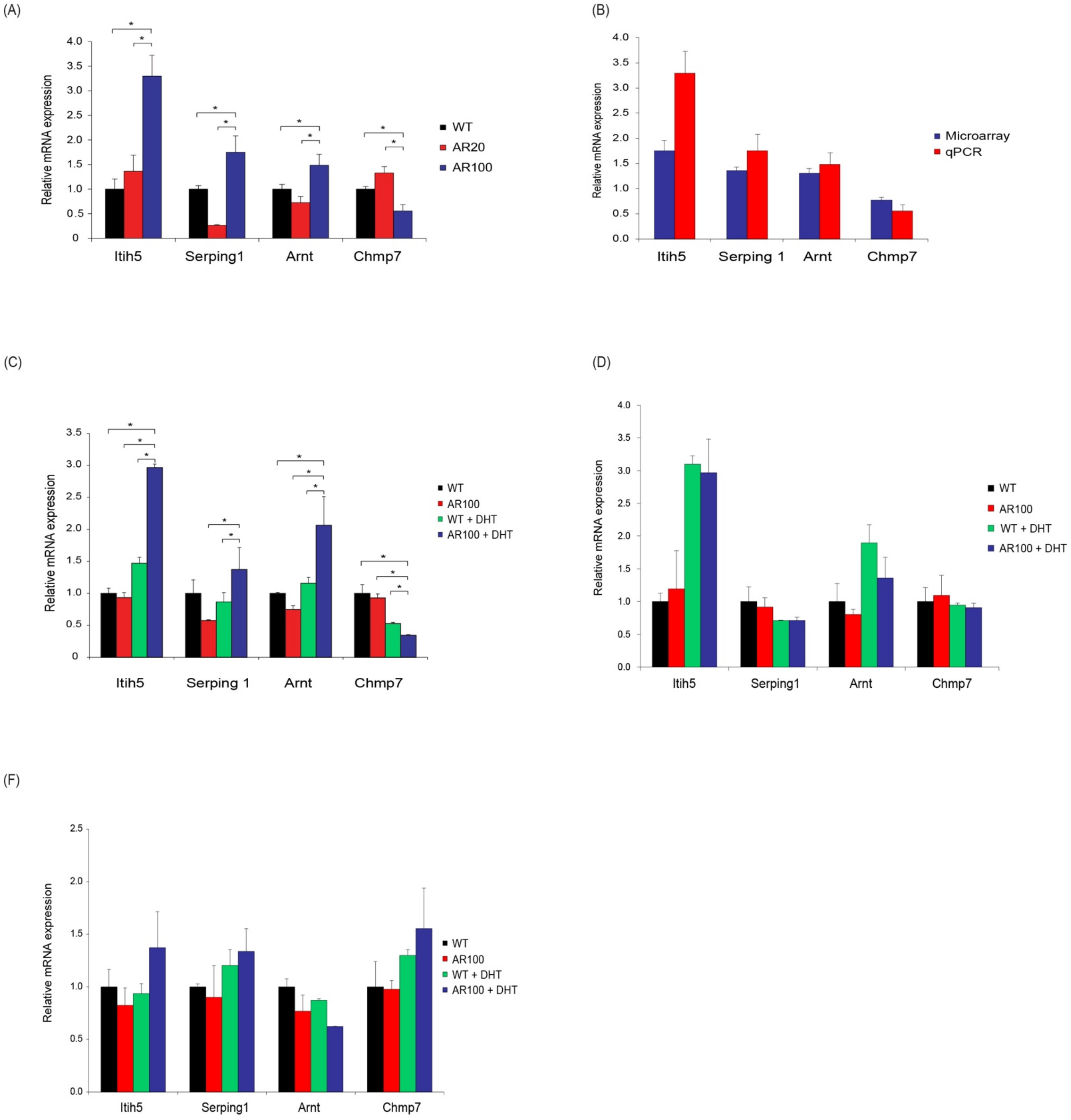
(A)
Relative mRNA expression
0
0.5
1.0
1.5
2.0
2.5
3.0
3.5
4.0
*
WT
AR20
AR100
Itih5
Serping1
Arnt
Chmp7
(B)
Relative mRNA expression
0.0
0.5
1.0
1.5
2.0
2.5
3.0
3.5
4.0
Microarray
qPCR
Itih5
Serping 1
Arnt
Chmp7
(C)
Relative mRNA expression
0
0.5
1.0
1.5
2.0
2.5
3.0
3.5
WT
AR100
WT + DHT
AR100 + DHT
Itih5
Serping 1
Arnt
Chmp7
(D)
Relative mRNA expression
0.0
0.5
1.0
1.5
2.0
2.5
3.0
3.5
4.0
WT
AR100
WT + DHT
AR100 + DHT
Itih5
Serping1
Arnt
Chmp7
(F)
Relative mRNA expression
0.0
0.5
1.0
1.5
2.0
2.5
WT
AR100
WT + DHT
AR100 + DHT
Itih5
Serping1
Arnt
Chmp7

Fig. S2

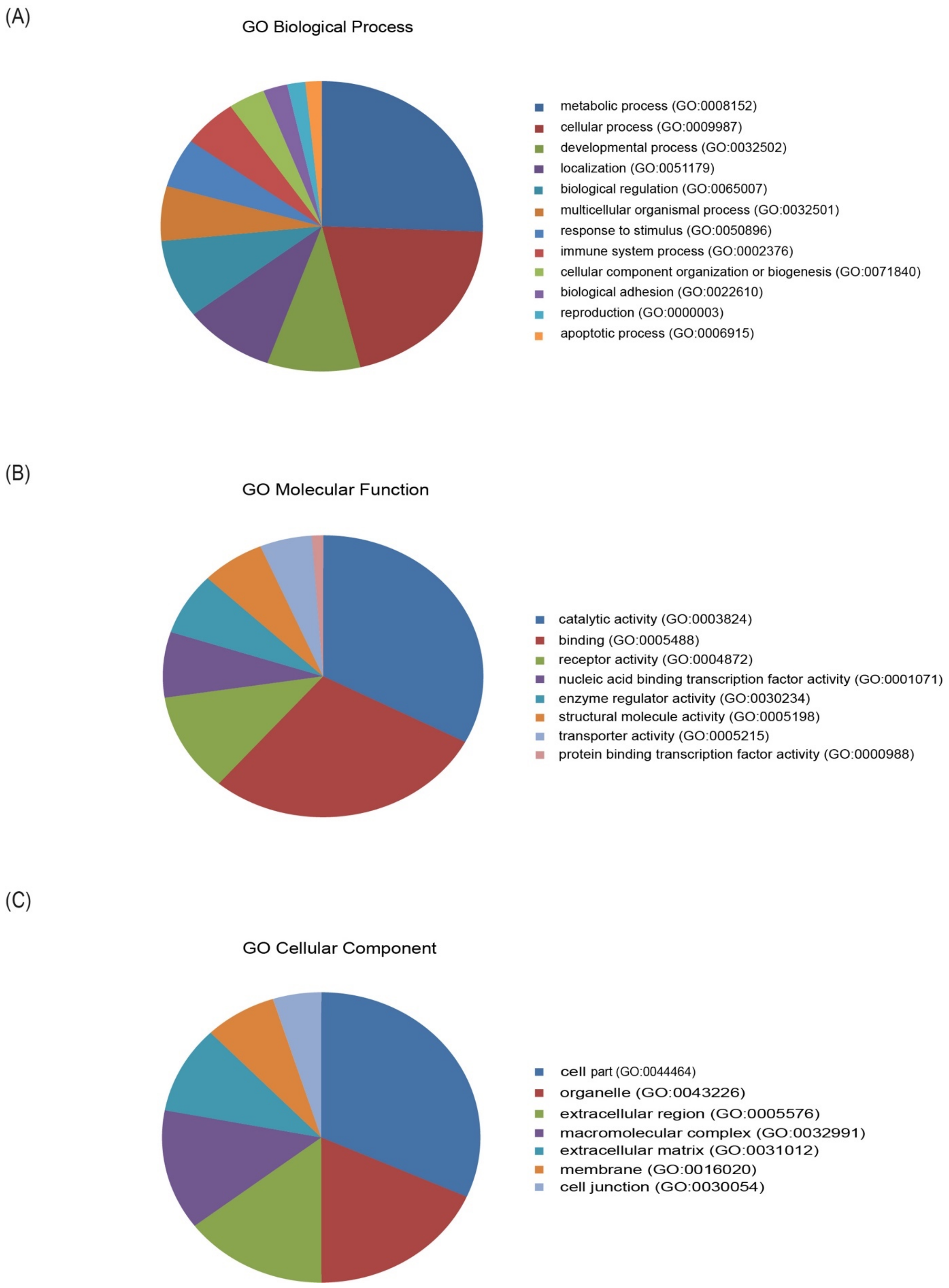
(A)
GO Biological Process
metabolic process (GO:0008152)
cellular process (GO:0009987)
developmental process (GO:0032502)
localization (GO:0051179)
biological regulation (GO:0065007)
multicellular organismal process (GO:0032501)
response to stimulus (GO:0050896)
immune system process (GO:0002376)
cellular component organization or biogenesis (GO:0071840)
biological adhesion (GO:0022610)
reproduction (GO:0000003)
apoptotic process (GO:0006915)
(B)
GO Molecular Function
catalytic activity (GO:0003824)
binding (GO:0005488)
receptor activity (GO:0004872)
nucleic acid binding transcription factor activity (GO:0001071)
enzyme regulator activity (GO:0030234)
structural molecule activity (GO:0005198)
transporter activity (GO:0005215)
protein binding transcription factor activity (GO:0000988)
(C)
GO Cellular Component
cell part (GO:0044464)
organelle (GO:0043226)
extracellular region (GO:0005576)
macromolecular complex (GO:0032991)
extracellular matrix (GO:0031012)
membrane (GO:0016020)
cell junction (GO:0030054)

Fig. S3. (A) ClueGO protein-protein interaction network

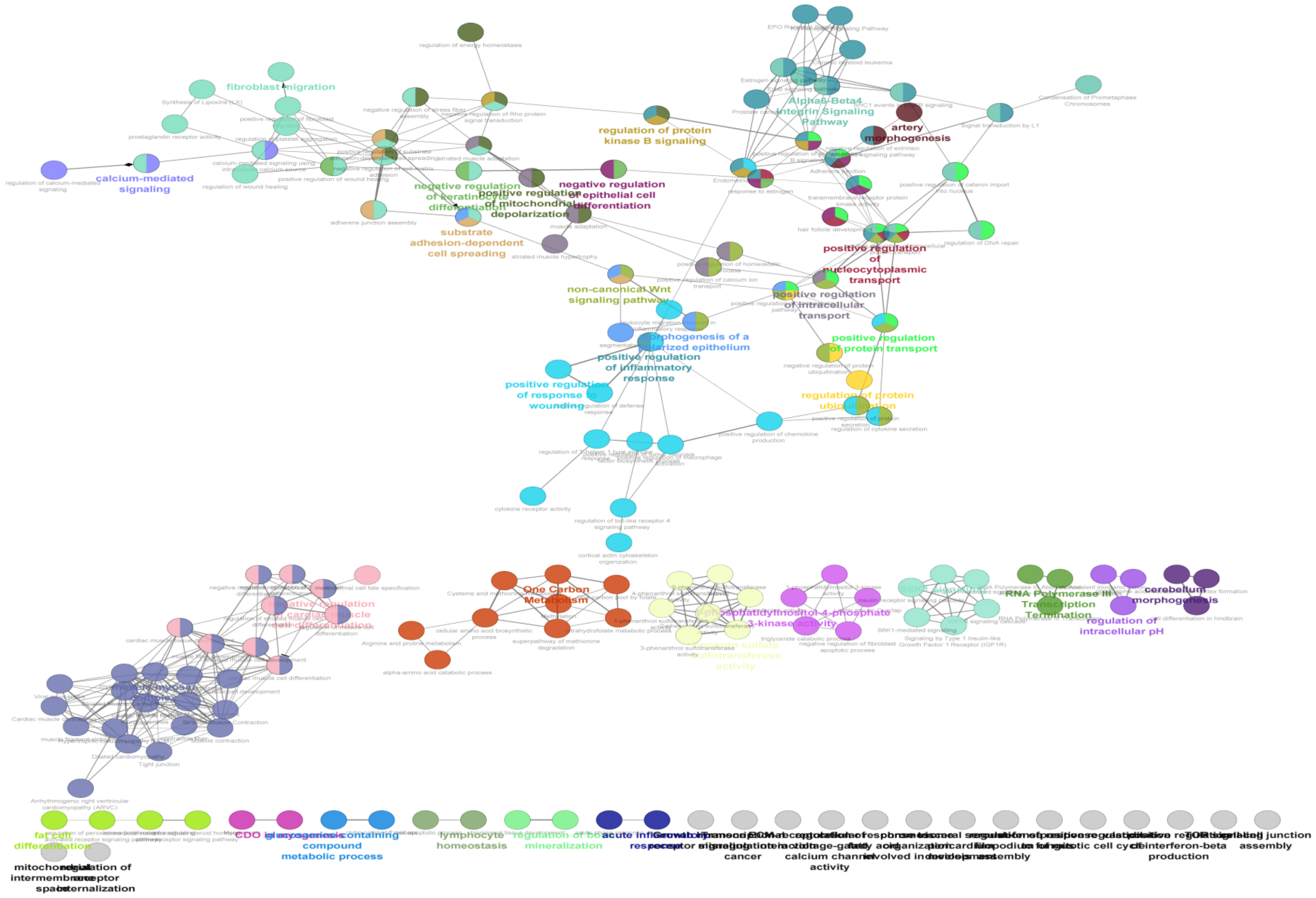

Fig. S3. (B) CluePedia enrichment of ClueGO protein-protein interaction network

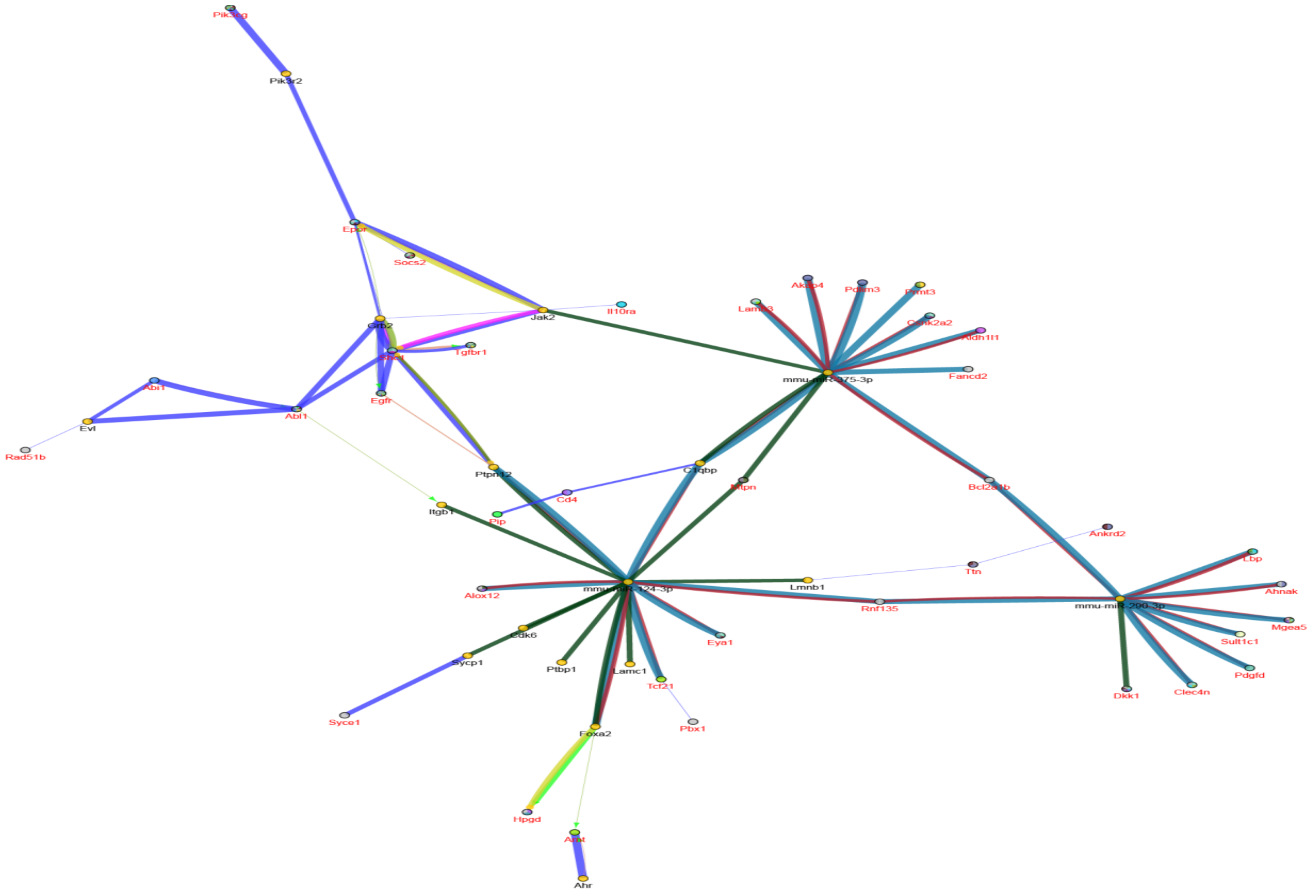

Fig. S4. (A) GSEA p53 pathway analysis

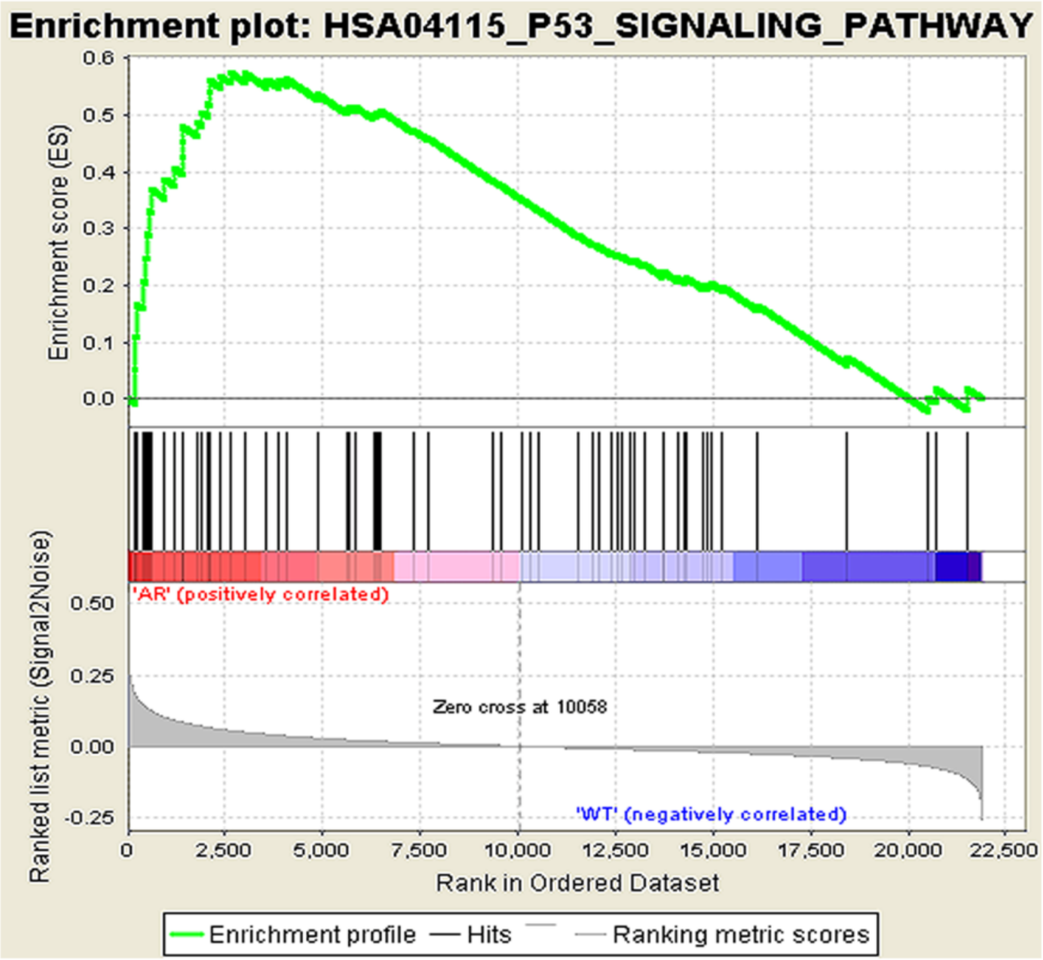

Fig. S4. (B) KEGG p53 pathway

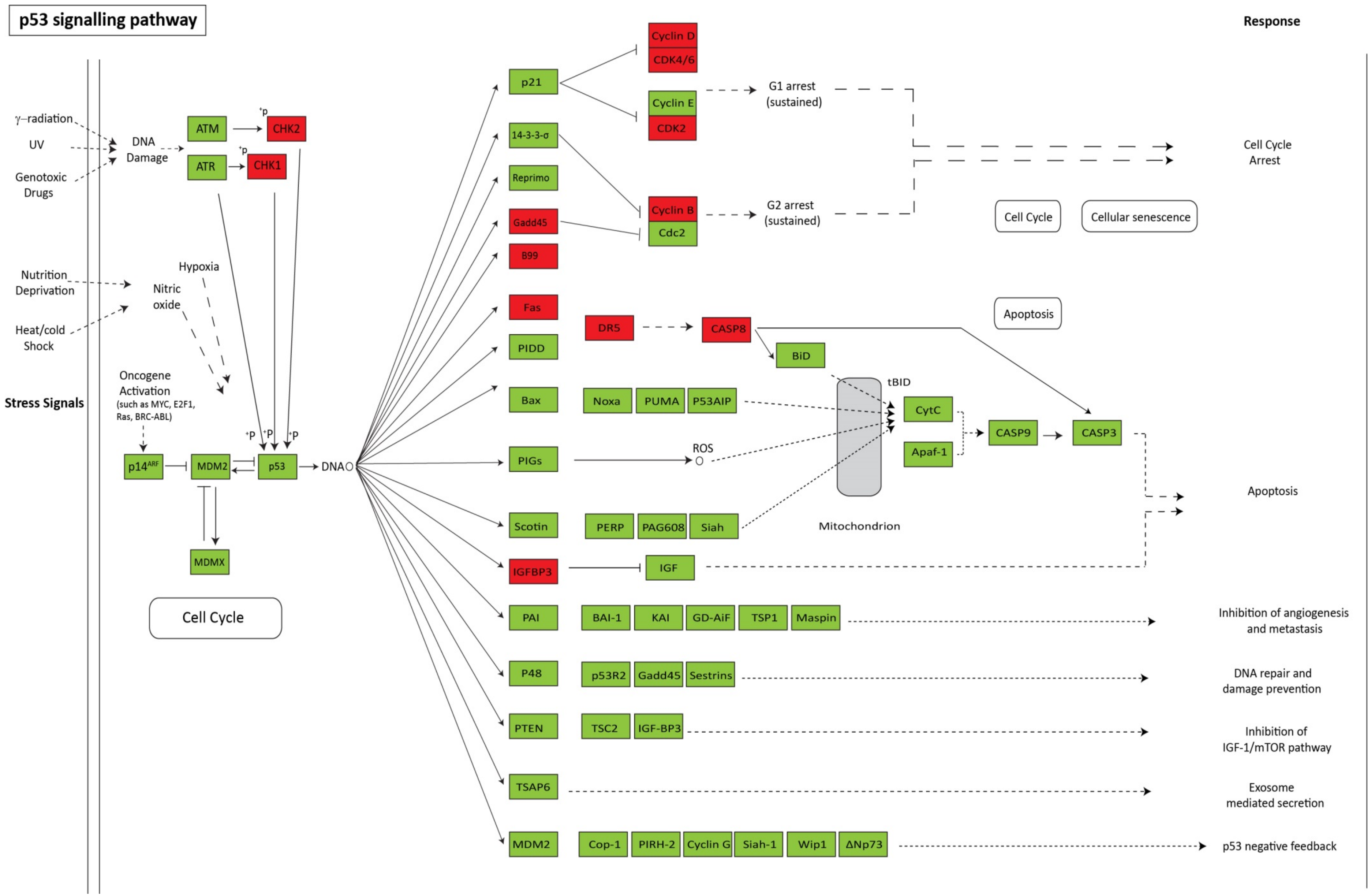

Fig. S4. (C) KEGG Lysosome

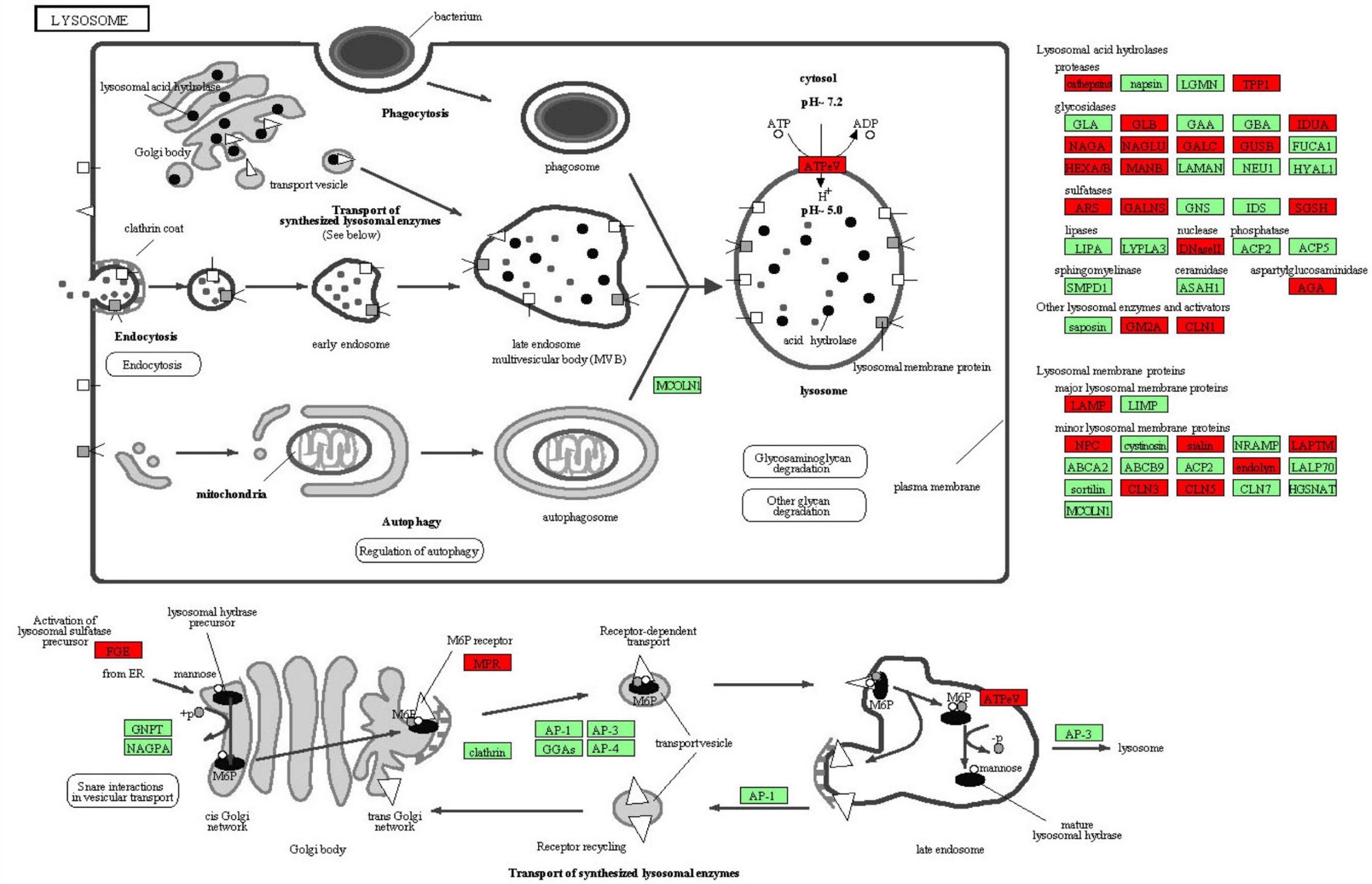

Fig. S5

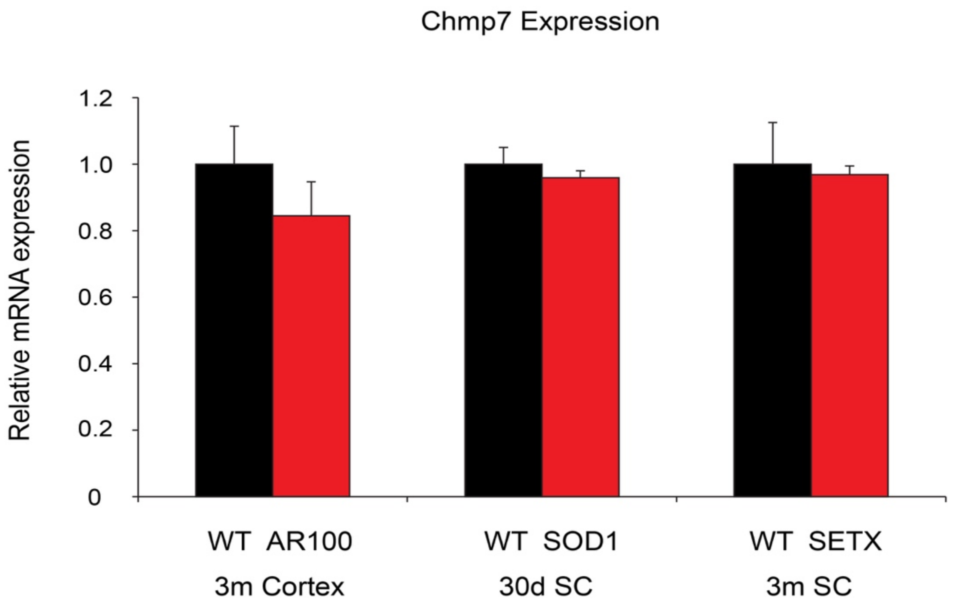
Chmp7 Expression
Relative mRNA expression
1.2
1.0
0.8
0.6
0.4
0.2
0
WT AR100
3m Cortex
WT SOD1
30d SC
WT SETX
3m SC

Fig. S6

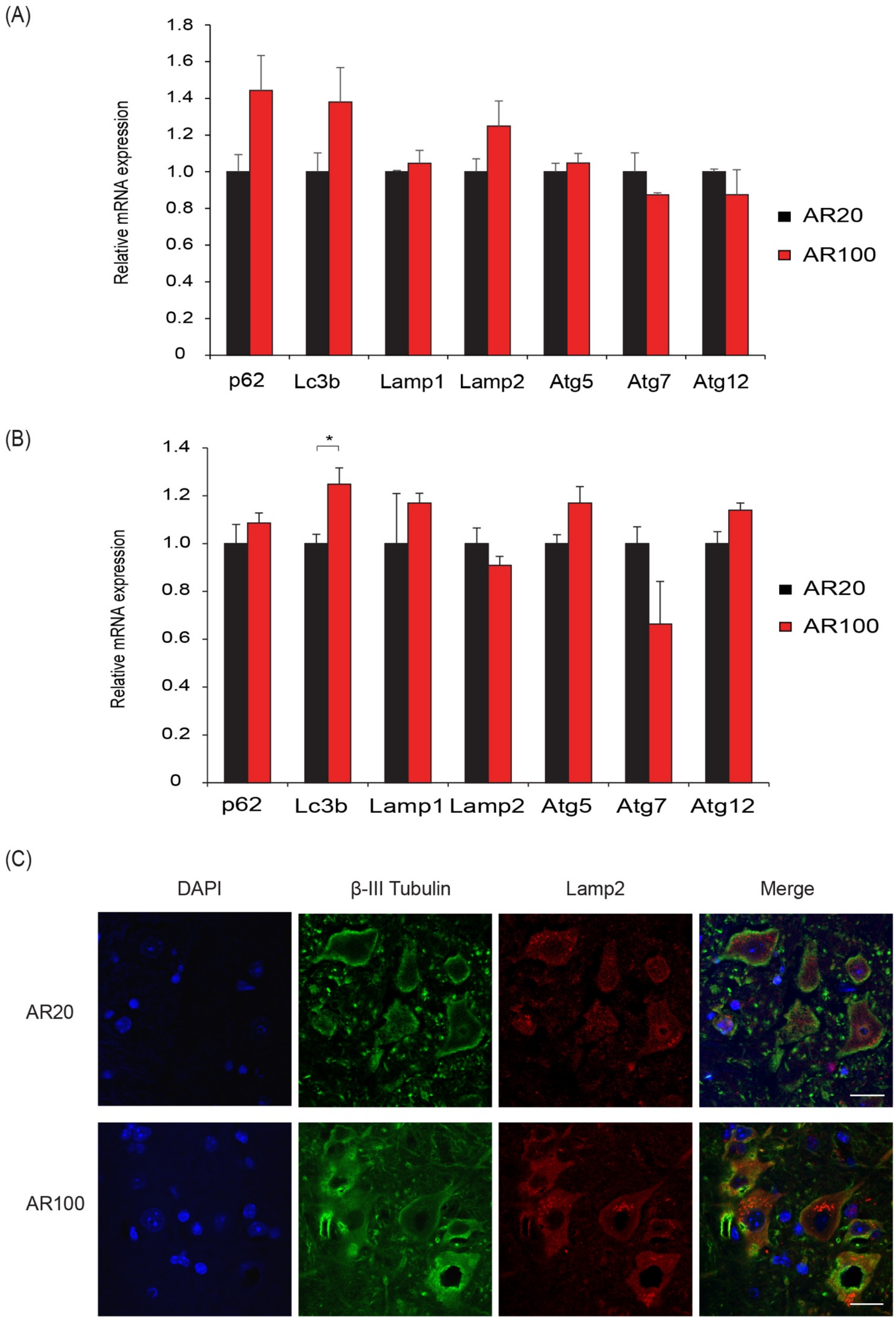
(A)
Relative mRNA expression
1.8
1.6
1.4
1.2
1.0
0.8
0.6
0.4
0.2
0
p62
Lc3b
Lamp1
Lamp2
Atg5
Atg7
Atg12
AR20
AR100
(B)
*
Relative mRNA expression
1.4
1.2
1.0
0.8
0.6
0.4
0.2
0
p62
Lc3b
Lamp1
Lamp2
Atg5
Atg7
Atg12
AR20
AR100
(C)
DAPI
β-III Tubulin
Lamp2
Merge
AR20
AR100

Fig. S7.

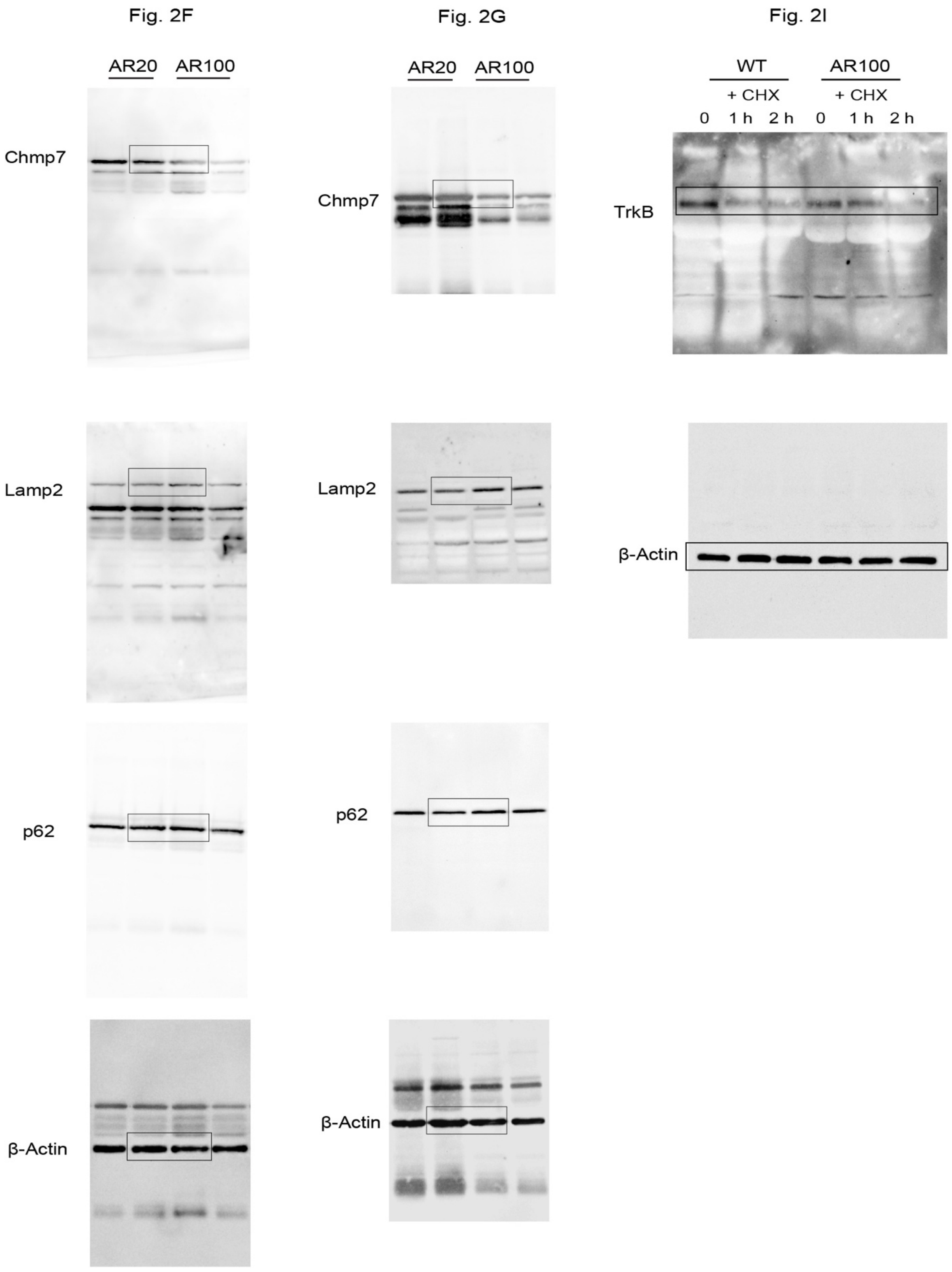
Fig. 2F
AR20
AR100
Chmp7
Lamp2
p62
β-Actin
Fig. 2G
AR20
AR100
Chmp7
Lamp2
p62
β-Actin
Fig. 2I
WT
AR100
+ CHX
+ CHX
0
1 h
2 h
0
1 h
2 h
TrkB
β-Actin

Fig. S8.

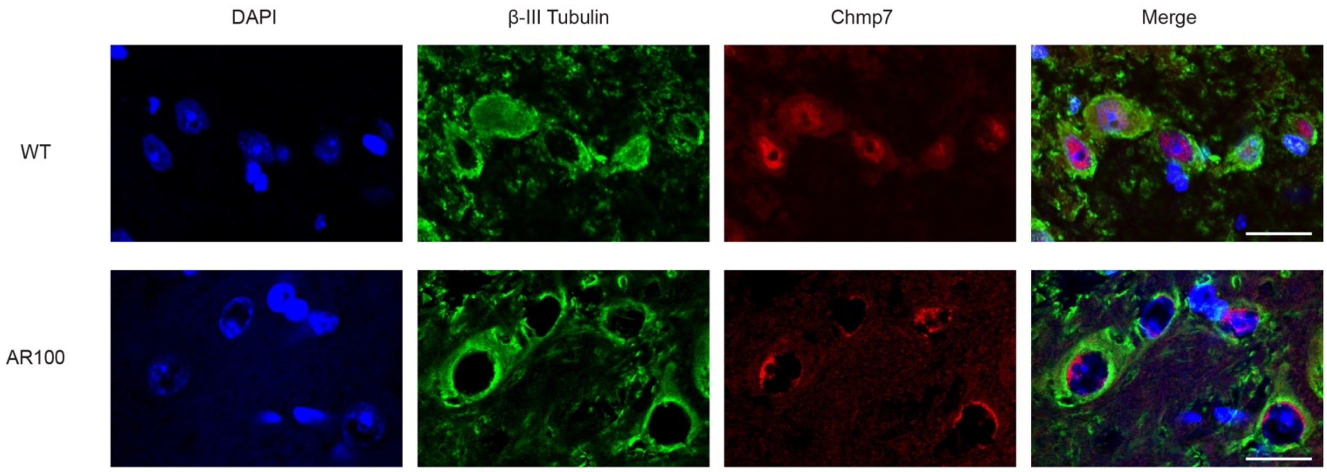

Supplement: Supplementary file 1 — Supplementary Information [file 41598_2019_40118_MOESM1_ESM.pdf]
